# The Effect of Commercial Genetic Selection on Somatotropic Gene Expression in Broilers: A Potential Role for Insulin-Like Growth Factor Binding Proteins in Regulating Broiler Growth and Body Composition

**DOI:** 10.3389/fphys.2022.935311

**Published:** 2022-06-27

**Authors:** Lauren A. Vaccaro, Tom E. Porter, Laura E. Ellestad

**Affiliations:** ^1^ Department of Poultry Science, University of Georgia, Athens, GA, United States; ^2^ Department of Animal and Avian Sciences, University of Maryland, College Park, MD, United States

**Keywords:** somatotropic axis, growth, insulin-like growth factor, insulin-like growth factor binding protein, endocrine signaling, paracrine signaling, broiler, Athens-Canadian Random Bred

## Abstract

The somatotropic axis influences growth and metabolism, and many of its effects are a result of insulin-like growth factor (IGF) signaling modulated by IGF-binding proteins (IGFBPs). Modern commercial meat-type (broiler) chickens exhibit rapid and efficient growth and muscle accretion resulting from decades of commercial genetic selection, and it is not known how alterations in the IGF system has contributed to these improvements. To determine the effect of commercial genetic selection on somatotropic axis activity, two experiments were conducted comparing legacy Athens Canadian Random Bred and modern Ross 308 male broiler lines, one between embryonic days 10 and 18 and the second between post-hatch days 10 and 40. Gene expression was evaluated in liver and breast muscle (*pectoralis major*) and circulating hormone concentrations were measured post-hatch. During embryogenesis, no differences in IGF expression were found that corresponded with difference in body weight between the lines beginning on embryonic day 14. While hepatic IGF expression and circulating IGF did not differ between the lines post-hatch, expression of both *IGF1* and *IGF2* mRNA was greater in breast muscle of modern broilers. Differential expression of select IGFBPs suggests their action is dependent on developmental stage and site of production. Hepatic *IGFBP1* appears to promote embryonic growth but inhibit post-hatch growth at select ages. Results suggest that local IGFBP4 may prevent breast muscle growth during embryogenesis but promote it after hatch. Post-hatch, *IGFBP2* produced in liver appears to inhibit body growth, but IGFBP2 produced locally in breast muscle facilitates development of this tissue. The opposite appears true for IGFBP3, which seems to promote overall body growth when produced in liver and restrict breast muscle growth when produced locally. Results presented here suggest that paracrine IGF signaling in breast muscle may contribute to overall growth and muscle accretion in chickens, and that this activity is regulated in developmentally distinct and tissue-specific contexts through combinatorial action of IGFBPs.

## Introduction

Growth and body composition in vertebrates are controlled by several highly conserved endocrine axes (Levine, 2012; Vaccaro et al., 2021). In particular, the somatotropic axis is known to regulate growth and development of mammals *via* cellular proliferation and metabolic effects in muscle, bone, and adipose tissue (Clark and Robinson, 1996; Gahete et al., 2016). However, its physiological impact on these processes is not as well understood in birds. Particularly lacking is information regarding how local production of insulin-like growth factor (IGF) 1 and IGF2 in tissues such as muscle impacts growth and body composition and how IGF-binding proteins (IGFBPs) regulate both endocrine and paracrine IGF signaling.

The key effector hormones in the somatotropic axis include IGF1 and IGF2 (Stewart and Rotwein, 1996), which are synthesized in the liver upon growth hormone receptor (GHR) activation (Kajimoto and Rotwein, 1989; Dewil et al., 1999; Herrington and Carter-Su, 2001; Woelfle et al., 2005; Brooks et al., 2008). A dwarf phenotype is observed in chickens deficient in GHR signaling (Hutt, 1959; Burnside et al., 1992; Chen et al., 2009), and this is partially caused by decreased hepatic IGF production (Burnside and Cogburn, 1992). On the cellular level, IGFs downregulate apoptosis while increasing cellular proliferation by binding the type 1 IGF receptor (IGFR1) (Girbau et al., 1989; Duclos and Goddard, 1990; D’Costa et al., 1998). This would imply a direct relationship between IGF signaling and growth in chickens, but studies have been inconclusive. Direct IGF1 administration did not stimulate growth in two to three week-old male chickens (McGuinness and Cogburn, 1991; Czerwinski et al., 1998) or four week-old females (Huybrechts et al., 1992). Increased hepatic *IGF1* mRNA expression has been observed in chickens selected for high body weight (Beccavin et al., 2001), but not consistently (Giachetto et al., 2004). Similarly, fast-growing chickens had greater plasma IGF2 (Scanes et al., 1989), but IGF2 did not induce weight gain when directly administered (Buyse and Decuypere, 1999). Studies investigating levels of growth hormone (GH), which is classically thought to induce IGF secretion from the liver, also yield results inconsistent with the idea that increased somatotropic activity always leads to increased growth. Pituitary GH expression was greater between 3 and 7 weeks of age in male broilers with lower body weight as compared to those with a higher body weight (Ellestad et al., 2019), and the percentage of GH-secreting cells in slow-growing chickens was greater at 5 weeks of age, though fast-growing embryos secreted more GH per hour (Porter, 1998). Circulating GH was also found to be higher in chickens selected for egg production (layers) than those selected for meat production (broilers), despite layers growing slower and having lower body weights (Reiprich et al., 1995).

Cellular effects induced by IGF signaling are regulated by IGFBPs. These proteins are highly conserved across vertebrates (Armstrong et al., 1989; Allander et al., 1995; Schoen et al., 1995; Allander et al., 1997; Kelley et al., 2002), although IGFBP6 has not been retained in birds. Growth modulation occurs when an IGFBP physically binds an IGF to enhance or reduce receptor affinity, extend the hormone’s half-life, or alter its tissue specificity (Baxter, 1991; Kim, 2010). For example, *IGFBP1* inhibits protein synthesis in skeletal muscle (Frost and Lang, 1999), while IGFBP2 and IGFBP4 inhibit long bone growth (Mohan et al., 1995; Fisher et al., 2005). In myoblasts, IGFBP5 has a proliferative effect when bound to IGF1 but an inhibitory effect upon binding IGF2 (Ewton et al., 1998). Additionally, some IGFBPs can act independently. For example, IGFBP2 can upregulate apoptosis (Schutt et al., 2004; Klaus et al., 2006), while IGFBP5 can enhance bone cell proliferation (Mohan et al., 1995). As both ligand-dependent and ligand-independent effects of IGFBPs are important in growth regulation, their actions may contribute to the enhanced growth and muscle accretion of commercial modern broiler chickens.

Commercial modern broilers are raised specifically for meat production and have an increased growth rate, greater body weight, reduced feed conversion ratio (FCR; g feed intake/g body weight gain), and higher meat yields (Bartov, 1982; Goddard et al., 1988; Havenstein et al., 1994; Berrong and Washburn, 1998; Havenstein et al., 2003; Collins et al., 2014), all of which are the result of decades of artificial genetic selection by the poultry industry. A useful experimental model to investigate the impact of the somatotropic axis on broiler growth and body composition is the comparison of commercially selected broilers currently used by the poultry industry with non-selected ones. Athens Canadian Random Bred (ACRB) legacy broilers are representative of slower-growing, lower body weight birds prior to the beginning of intensive commercial broiler selection (Hess, 1962; Collins et al., 2014; Marks et al., 2016). Administration of a current commercial-type diet to ACRBs reduced their FCR some but not to the point of a commercial broiler and did not increase growth or body weight (Havenstein et al., 1994), which makes them an ideal genetic control strain. In a recent study where ACRB were compared with Ross 308 commercial broilers to identify effects of commercial genetic selection on the corticotropic and thyrotropic axes, it was reported that Ross 308 body weights were significantly greater than those for ACRB beginning during the last week of embryogenesis, and this difference continued throughout juvenile development (Vaccaro et al., 2021). FCR of ACRB was also significantly higher than of Ross 308, reflecting the improved efficiency of feed nutrient use in commercial modern broilers. Together, these results suggest that physiological changes induced by commercial genetic selection begin to appear mid-embryogenesis. Given the conservation of the somatotropic axis across species and its importance in mediating tissue growth and development in mammals, it is likely that IGFs, their receptors, and IGFBPs are linked to improvements in commercial modern broiler growth efficiency. Therefore, the objective of this study was to determine the effect of commercial genetic selection on mRNA expression and circulating hormone concentrations within the somatotropic axis by comparing these parameters between Ross 308 and legacy ACRB broiler lines.

## Materials and Methods

### Animals and Tissue Collection

Samples used for this study were collected from male ACRB and Ross 308 broilers during the same two experiments described in a previously published study (Vaccaro et al., 2021). The first experiment was conducted during embryogenesis, and the second was conducted during post-hatch juvenile development. All experimental procedures using animals were conducted in accordance with University of Georgia and University of Maryland Institutional Animal Care and Use guidelines.

In the first experiment, skin, liver, and breast muscle (*p. major*) were collected from 12 embryos of each line on embryonic days (e) 10, 12, 14, 16, and 18, with e0 being the day eggs were placed in the incubator. Eggs from both lines were co-incubated in the same incubator under identical conditions. The sex of each embryo was determined by PCR analysis of the sexually dimorphic chromo-helicase-DNA binding protein (Fridolfsson and Ellegren, 1999) using genomic DNA extracted from skin tissue, as previously described (Vaccaro et al., 2021). Liver and breast muscle from four male embryos of each line at each age (*n* = 4) were used for gene expression analysis as described below.

In the second experiment, males of each line were raised in separate floor pens (*n* = 8 floor pens per line) within one room, so that environmental conditions were identical. Both lines had free access to water and the same three-phase modern commercial-type diet as previously described (Vaccaro et al., 2021). Liver, breast muscle (*P. major*), and plasma were collected from one bird per pen (*n* = 8 per line) on post-hatch days (d) 10, 20, 30, and 40 as previously described (Vaccaro et al., 2021). Briefly, liver and breast muscle were immediately snap-frozen in liquid nitrogen and stored at −80°C prior to being used for gene expression analysis. Whole blood was collected into syringes coated with lithium heparin and stored on ice for no longer than 60 min prior to isolation of plasma by centrifugation at 1,500x *g* and 4°C for 10 min. Plasma was stored at −20°C prior to use for evaluation of circulating hormone levels, as described below.

### Reverse Transcription-Quantitative PCR (RT-qPCR)

Total RNA was isolated from liver and breast muscle using RNeasy Mini kits (Qiagen) with modifications for lipid-rich or fibrous tissues, respectively, and analyzed by RT-qPCR as previously described (Vaccaro et al., 2021). Briefly, total RNA (1 µg) was reverse transcribed with random hexamer primers (ThermoFisher Scientific, Waltham, MA, United States) and M-MuLV reverse transcriptase (New England Biolabs, Ipswich, MA, United States). Resulting cDNA was amplified by qPCR using intron-spanning primers (Table 1; Integrated DNA Technologies, Coralville, IA, United States) designed with Primer Express software (Applied Biosystems, Foster City, CA, United States). Serial dilutions of pooled liver and muscle cDNA were analyzed by qPCR to determine amplification efficiency for each primer pair, which was calculated using the following equation: efficiency = [10 ^(−1/slope)^−1] (Livak and Schmittgen, 2001; Rutledge and Stewart, 2008).

**TABLE 1:** Primers used for reverse transcription-quantitative PCR.

Gene symbol	Forward primer (5′-3′)	Reverse primer (5′-3′)	Transcript ID^1^	Efficiency
IGFs				
*IGF1*	TGA​GCT​GGT​TGA​TGC​TCT​TC	AGC​CTC​CTC​AGG​TCA​CAA​CT	20816	0.99
*IGF2*	AGT​CAG​AGC​GTG​ACC​TCT​CC	CTG​CGA​GCT​CTT​CTT​CTG​C	53800	1.05
Hormone receptors				
*GHR*	TGC​TGA​TTT​TTC​CTC​CTG​TG	GGC​TGG​CTA​AGA​TGG​AGT​TC	23973	1.08
*IGF1R*	TGG​GGA​CCT​CAA​AAG​TTA​CC	ATC​CCA​TCA​GCA​ATC​TCT​CC	74990	1.04
Hormone binding proteins				
*IGFBP1*	CAG​AGA​AGT​GGA​GGG​GAC​AT	CTT​CTG​GGG​ATC​CAG​GAA​T	47713	
*IGFBP2*	ATC​ACA​ACC​ACG​AGG​ACT​CA	GAG​GGA​GTA​GAG​GTG​CTC​CA	18698	0.96
*IGFBP3*	TTG​AGT​CCT​AGG​GGT​TTC​CA	ATA​TCC​AGG​AAG​CGG​TTG​TC	82156	1.02
*IGFBP4*	AACTTCCACCCCAAGCAG	AAT​CCA​AGT​CCC​CCT​TCA​G	68153	0.96
*IGFBP5*	CTG​AAG​AGC​AGC​CAG​AGG​AT	TTG​TCC​ACA​CAC​CAA​CAC​AG	38163	0.98
*IGFBP7*	ATG​TGA​CAG​GAG​CAC​AGA​TCT​ACC​T	TCT​GGA​TAC​CAT​ACT​GTC​CTC​GAA​T	61018	0.95
Reference genes				
*GAPDH*	AGC​CAT​TCC​TCC​ACC​TTT​GAT	AGT​CCA​CAA​CAC​GGT​TGC​TGT​AT	23323	1.00
*18s* ^2^	AGC​CTG​CGG​CTT​AAT​TTG​AC	CAA​CTA​AGA​ACG​GCC​ATG​CA	173612	0.96

1 Transcript identification from Ensembl chicken genome assembly GRCg6a (http://www.ensembl.org/Gallus_gallus/Info/Index) preceded by ENSGALT000000.

2 Sequence for 18S rRNA, is not on the assembled chicken genome, and primers were designed based on the sequence in GenBank (Accession Number AF173612).

Transcripts in liver were normalized to glyceraldehyde 3-phosphate dehydrogenase (*GAPDH*), and those in muscle were normalized to 18s ribosomal rRNA (18s rRNA). The equation (2^ΔCt^)_target_/(2^ΔCt^)_GAPDH or 18s_, where ΔCt = Ct_no RT_−CT_sample_, was used to transform and normalize data as previously described (Ellestad et al., 2009; Ellestad and Porter, 2013; Ellestad et al., 2015; Payne et al., 2019; Vaccaro et al., 2021). Each transcript’s line-by-age interactive data are expressed relative to the line and age with the highest mRNA level, and main effect data are expressed relative to the line or age with the highest mRNA level. As a result, the line-by-age, line, or age value with the highest expression level was 100% in all cases.

### Insulin-Like Growth Factor Enzyme-Linked Immunosorbent Assays

Samples were analyzed in duplicate on a VICTOR3 Multilabel Plate Reader (Perkin Elmer, Waltham, MA, United States) using commercially available competitive-binding ELISAs (Cusabio, Houston, TX, United States) for IGF1 and IGF2, which have sensitivity limits of 125 and 62.5 pg/ml, respectively. ELISAs were performed according to manufacturer’s instructions with the modification that plates were incubated for 18 h at 4°C instead of 60 min at 37°C after adding the standards or samples and biotinylated IGF. Intra and inter-assay coefficient of variations (CVs) for IGF1 ELISAs were determined to be 4.023 and 6.479, respectively. Intra and inter-assay coefficient of variations (CVs) for IGF2 ELISAs were determined to be 10.0 and 34.6, respectively.

### Statistical Analysis

Data were analyzed with a two-way analysis of variance (ANOVA) using the Fit Model Procedure of JMP Pro 14 (SAS Institute, Cary, NC, United States), with relative RT-qPCR data being log_2_-transormed prior to analysis. When ANOVA indicated a significant line-by-age effect, line effect, or age effect (*p* ≤ 0.05), *post hoc* multiple means comparisons were performed using the test of least significant difference. Main effect means were only calculated and analyzed when there was not a significant interaction (*p* > 0.05).

## Results

### Insulin-Like Growth Factor and Hormone Receptor Expression During Embryonic Development

**FIGURE 1:**
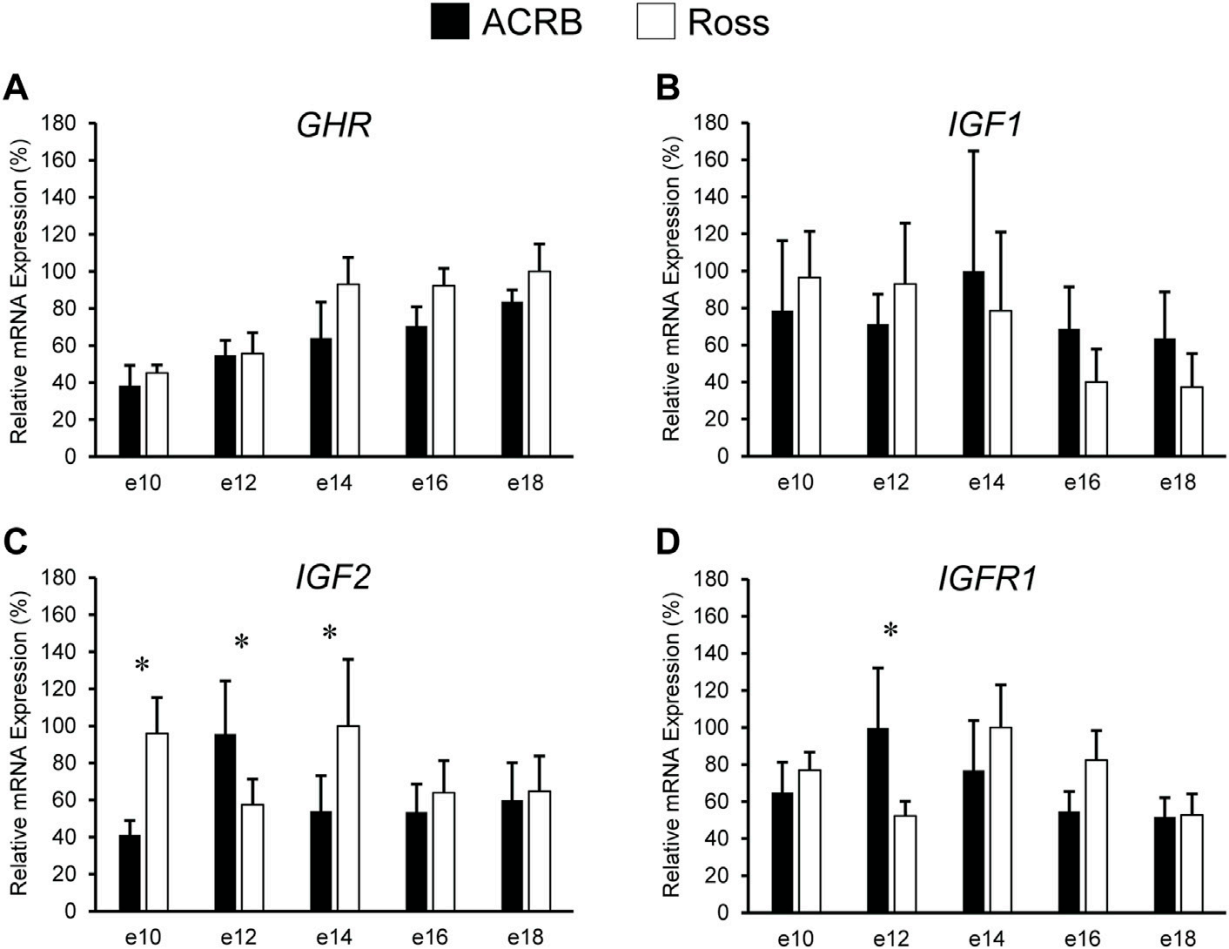
Relative mRNA expression of **(A)** *GHR*, **(B)** *IGF1*, **(C)** *IGF2*, and **(D)** *IGFR1* in liver on embryonic days e 10, 12, 14, 16, and 18 in legacy ACRB and modern Ross 308 male broilers. Relative expression levels were measured using RT-qPCR and normalized to *GAPDH* mRNA (*n* = 4 replicate birds per line at each age). The data (mean + SEM) are expressed relative to the line and age with the highest expression level (equivalent to 100%). No significant line-by-age interactions were detected for **(A)** *GHR* (*p* = 0.7777) or **(B)** *IGF1* (*p* = 0.7562), and main effect means for line and age for these genes are shown in Tables 2, 3, respectively. Significant line-by-age interactions were identified for **(C)** *IGF2* (*p* = 0.0003) and **(D)** *IGFR1* (*p* = 0.0235), and the presence of an asterisk (*) indicates a significant difference in expression between the lines at those ages (*p* ≤ 0.05).

Levels of mRNA for IGFs and somatotropic hormone receptors in embryonic ACRB and Ross liver are shown in Figure 1. Expression of *GHR* did not exhibit a significant line-by-age effect in embryonic liver (Figure 1A; *p* > 0.05), but a near significant main effect of line was observed in which Ross 308 had elevated expression as compared to ACRB (Table 2; *p* = 0.0640). A significant main effect of age for *GHR* was also detected in liver, with levels significantly and steadily increasing between e10 and e18 (Table 3; *p* ≤ 0.05). No significant differences in expression between lines or at different ages were detected for liver *IGF1* during embryogenesis (Figure 1B; Tables 2, 3; *p* > 0.05). Significant line-by-age interactive effects were detected for *IGF2* and *IGFR1* in liver, however. *IGF2* was approximately 2-fold greater in Ross on e10 and e14, but a transient decrease in expression in Ross on e12 with a concomitant increase in ACRB expression resulted in reduced levels of Ross *IGF2* at this age (Figure 1C; *p* ≤ 0.05). A similar though less prominent expression pattern was observed for liver *IGFR1*, with levels in ACRB being approximately two-fold greater than Ross on e12 (Figure 1D; *p* ≤ 0.05).

**TABLE 2:** Means^1^ (±SEM) and ANOVA *p*-values of the line main effect for somatotropic gene expression in embryonic male ACRB and Ross 308 broilers.

	ACRB	Ross 308	*p*-value
IGFs and Receptors			
Liver (%)^2^			
*GHR*	80.6 ± 7.6	100 ± 8.9	0.0640
*IGF1*	75.2 ± 14.9	100 ± 34.5	0.7004
Muscle (%)^2^			
*GHR*	100 ± 7.9	90.5 ± 7.4	0.3378
*IGF1*	93.1 ± 12.1	100 ± 9.9	0.7055
*IGF2*	100 ± 12.9	88.2 ± 13.5	0.4571
*IGF1R*	100 ± 10.9	84.5 ± 7.7	0.2150
IGFBPs			
Liver (%)^2^			
*IGFBP2*	95.5 ± 22.4	100 ± 17.7	0.6238
*IGFBP4*	87.3 ± 11.4	100 ± 14.7	0.3633
*IGFBP5*	100 ± 6.1	86.8 ± 5.8	0.0940
*IGFBP7*	82.1 ± 7.5	100 ± 12.4	0.2619
Muscle (%)^2^			
*IGFBP1*	99.8 ± 20.1	100 ± 15.8	0.7343
*IGFBP2*	100 ± 9.4	91.2 ± 5.6	0.6339
*IGFBP3*	100 ± 7.2	95.9 ± 6.1	0.6978
*IGFBP4*	100 ± 13.7^a^	69.7 ± 8.0^b^	0.0354
*IGFBP5*	100 ± 10.5	97.2 ± 8.9	0.8773
*IGFBP7*	100 ± 13.3	96.3 ± 10.0	0.7269

1 Means are only presented for data where a significant line-by-age interaction was not present and were calculated between embryonic day 10 and 18 for each line.

2 Data within each gene are expressed relative to the line with the highest mRNA, level (equal to 100%).

a,b Values within each gene that do not share a common letter are significantly different (*p* ≤ 0.05).

**TABLE 3:** Means^1^ (±SEM) and ANOVA *p*-values of the age main effect for somatotropic gene expression in embryonic male ACRB and Ross 308 broilers.

	e10	e12	e14	e16	e18	*p*-value
IGFs and Receptors						
Liver (%)^2^						
*GHR*	45.5 ± 6.1^c^	60.0 ± 7.0^bc^	85.5 ± 13.6^ab^	88.68 ± 8.3^ab^	100 ± 8.8^a^	0.0023
*IGF1*	51.3 ± 12.4	48.12 ± 10.2	100 ± 50.7	31.84 ± 8.4	29.59 ± 8.9	0.4101
Muscle (%)^2^						
*GHR*	56.5 ± 8.0^b^	82.66 ± 8.3^a^	100 ± 15.3^a^	92.8 ± 5.8^a^	81.3 ± 8.9^a^	0.0243
*IGF1*	71.8 ± 12.7^ab^	100 ± 8.9^a^	85.9 ± 14.6^ab^	54.1 ± 4.9^bc^	34.9 ± 2.3^c^	0.0006
*IGF2*	96.9 ± 15.9	86.2 ± 24.8	57.2 ± 13.8	96.2 ± 19.8	100 ± 19.3	0.4383
*IGF1R*	77.9 ± 8.9^abc^	81.6 ± 6.9^ab^	100 ± 15.9^a^	65.6 ± 12.9^bc^	54.5 ± 10.8^c^	0.0446
IGFBPs						
Liver (%)^2^						
*IGFBP2*	15.3 ± 2.1^c^	24.9 ± 4.7^c^	67.6 ± 15.5^b^	100 ± 27.9^ab^	98.0 ± 15.1^a^	<0.0001
*IGFBP4*	69.1 ± 11.0	72.3 ± 14.4	100 ± 26.3	51.9 ± 7.0	69.4 ± 13.8	0.5605
*IGFBP5*	68.2 ± 8.0^b^	80.2 ± 9.3^ab^	100 ± 7.5^a^	75.8 ± 6.5^b^	70.3 ± 4.9^b^	0.0271
*IGFBP7*	37.5 ± 4.7^c^	56.3 ± 8.2^b^	86.5 ± 15.5^a^	84.5 ± 13.7^a^	100 ± 7.9^a^	<0.0001
Muscle (%)^2^						
*IGFBP1*	100 ± 11.4^a^	58.9 ± 11.2^ab^	44.2 ± 26.0^bc^	36.7 ± 5.4^bc^	24.2 ± 4.6^c^	0.0068
*IGFBP2*	78.8 ± 5.7	64.5 ± 7.4	64.9 ± 7.20	100 ± 11.2	74.3 ± 10.6	0.0808
*IGFBP3*	90.2 ± 12.0	100 ± 6.7	85.1 ± 10.7	89.2 ± 10.6	81.9 ± 8.5	0.6923
*IGFBP4*	100 ± 16.9	76.4 ± 16.4	89.4 ± 26.8	75.2 ± 7.8	48.1 ± 8.6	0.0866
*IGFBP5*	100 ± 12.1	83.8 ± 9.3	82.9 ± 20.4	71.9 ± 6.9	73.6 ± 13.7	0.4908
*IGFBP7*	40.6 ± 8.8^c^	46.1 ± 3.3^c^	60.7 ± 11.2^bc^	78.8 ± 7.5^ab^	100 ± 15.1^a^	0.0009

1 Means are only presented for data where a significant line-by-age interaction was not present and were calculated across both lines at each embryonic day (e).

2 Data within each gene are expressed relative to the age with the highest mRNA level (equal to 100%).

a,b,c Values that do not share a common letter are significantly different (*p* ≤ 0.05).

**FIGURE 2:**
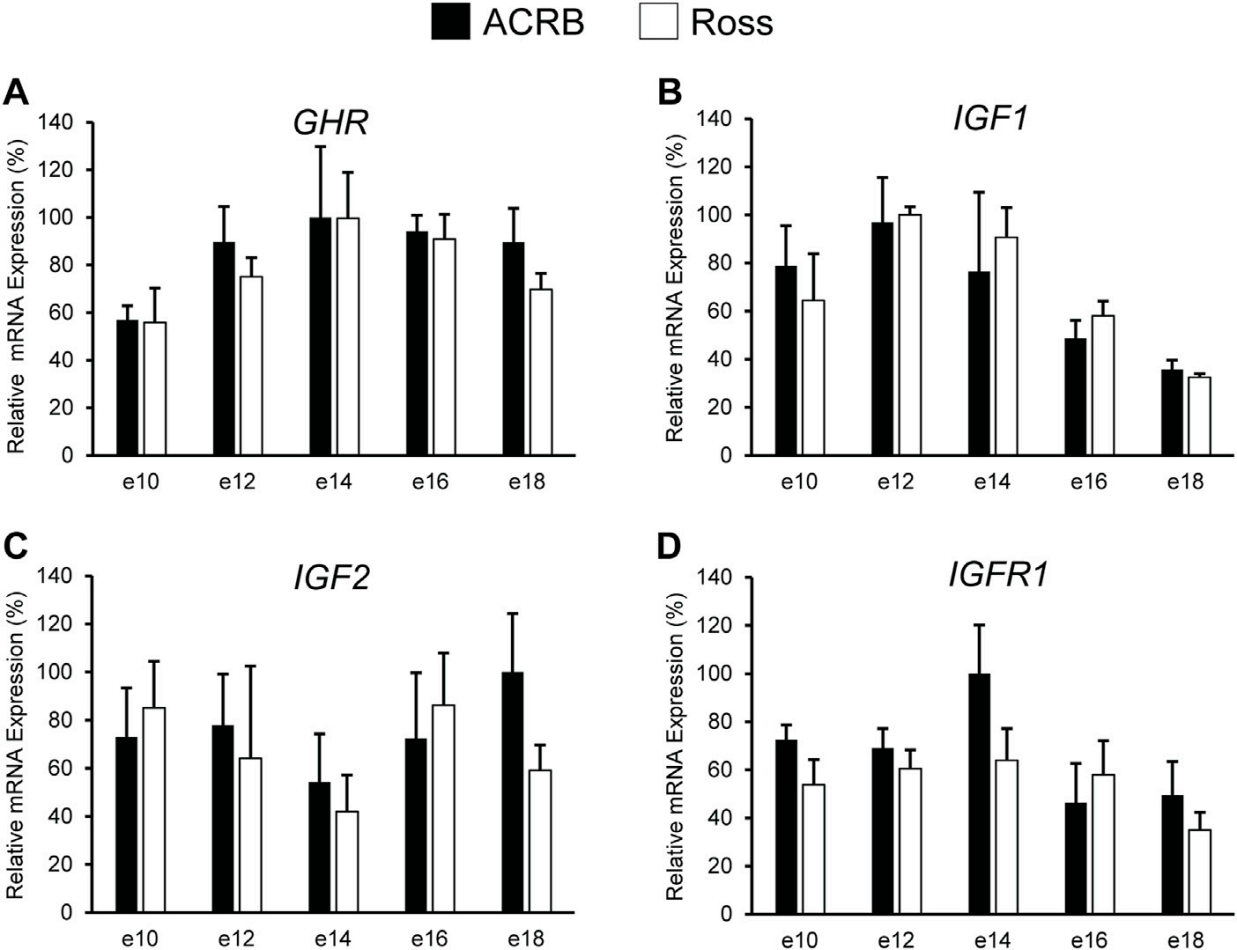
Relative mRNA expression of **(A)** *GHR*, **(B)** *IGF1*, **(C)** *IGF2*, and **(D)** *IGFR1* in breast muscle on embryonic days e 10, 12, 14, 16, and 18 in legacy ACRB and modern Ross 308 male broilers. Relative expression levels were measured using RT-qPCR and normalized to *18S* RNA (*n* = 4 replicate birds per line at each age). The data (mean + SEM) are expressed relative to the line and age with the highest expression level (equivalent to 100%). No significant line-by-age interactions were observed for **(A)** *GHR* (*p* = 0.9321), **(B)** *IGF1* (*p* = 0.5901), **(C)** *IGF2* (*p* = 0.6246), or **(D)** *IGF1R* (*p* = 0.4752), and main effect means of line and age all genes are presented in Tables 2, 3, respectively.

As shown in Figure 2, no significant line-by-age interactions were detected for any of these genes in embryonic breast muscle (Figures 2A–D; *p* > 0.05). However, *GHR*, *IGF1*, and *IGFR1* exhibited age main effects in this tissue (Table 3; *p* ≤ 0.05). Expression of *GHR* increased in both lines between e10 and e14 and remained elevated thereafter (Table 3; *p* ≤ 0.05). Expression of *IGF1* began to significantly decrease at e18 (Table 3; *p* ≤ 0.05). Expression of *IGFR1* dropped between e14 and 16 and remained low on e18 (Table 3; *p* ≤ 0.05). No main effect of age for *IGF2* was observed in breast muscle (Table 3; *p* > 0.05).

### Insulin-Like Growth Factor and Hormone Receptor Expression During Post-Hatch Development

**FIGURE 3:**
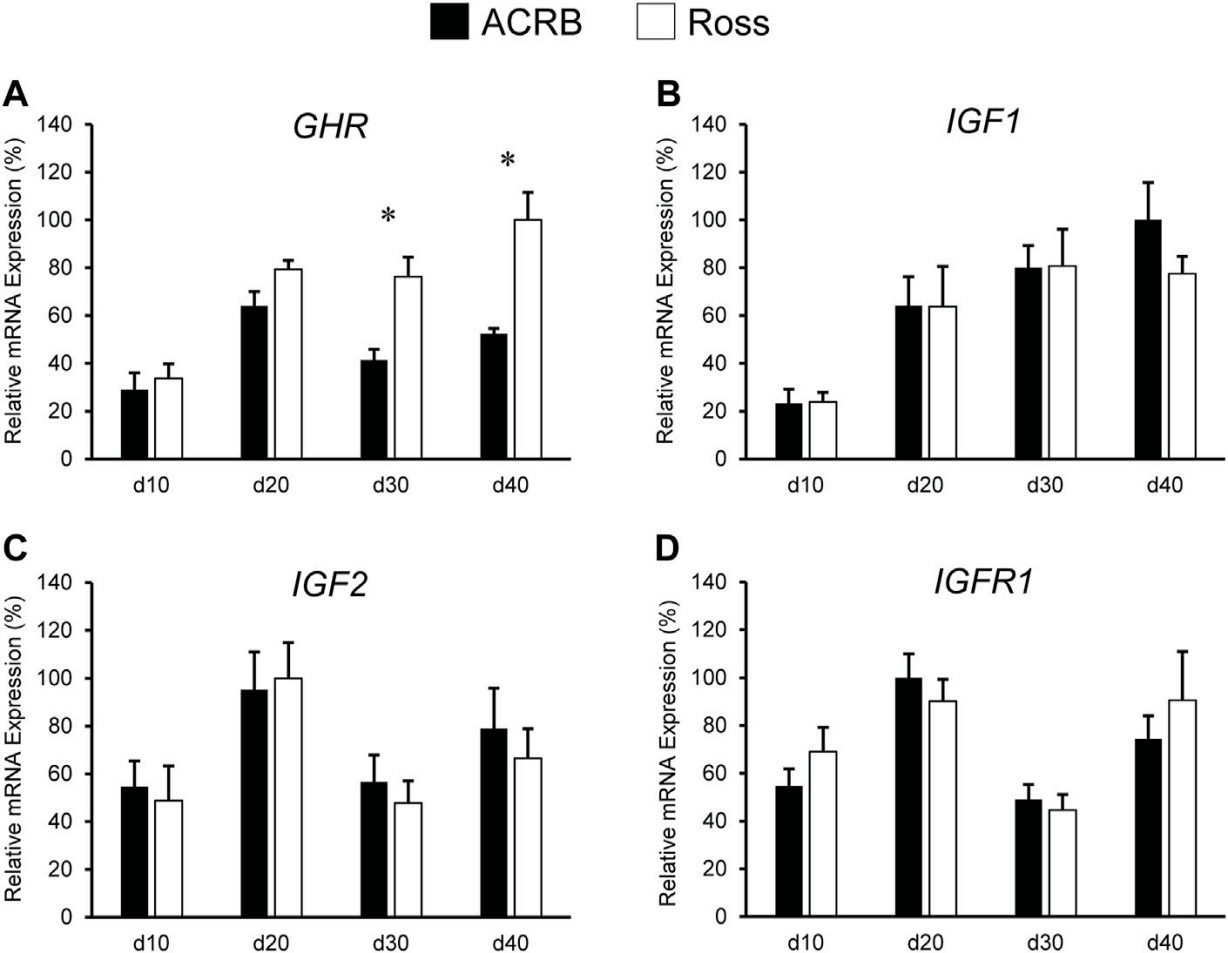
Relative mRNA expression of **(A)** *GHR*, **(B)** *IGF1*, **(C)** *IGF2*, and **(D)** *IGFR1* in liver on post-hatch days d 10, 20, 30, and 40 in legacy ACRB and modern Ross 308 male broilers. Relative expression levels were measured using RT-qPCR and normalized to *GAPDH* mRNA (*n* = 8 replicate birds per line at each age). The data (mean + SEM) are expressed relative to the line and age with the highest expression level (equivalent to 100%). A significant line-by-age interaction was detected for **(A)** *GHR* (*p* = 0.0446), and the presence of an asterisk (*) indicates a significant difference in expression between the lines at the indicated age. No significant line-by-age interactions were detected for **(B)** *IGF1* (*p* = 0.6890), **(C)** *IGF2* (*p* = 0.8688), or **(D)** *IGFR1* (*p* = 0.7405), and main effect means of line and age for these genes are presented in Tables 4, 5, respectively.

Expression levels of somatotropic hormones and receptors in ACRB and Ross post-hatch liver are presented in Figure 3. Only *GHR* exhibited a significant line-by-age interaction, in which expression was two-fold greater in Ross liver at both d30 and d40 (Figure 3A; *p* ≤ 0.05). No line-by-age interactions or main effects of line were observed *IGF1*, *IGF2*, or *IGFR1* (Figures 3B–D; *p* > 0.05), but they exhibited main age effects (Tables 2, 3; *p* ≤ 0.05). Expression of *IGF1* in both Ross and ACRB liver increased steadily between d10 and d30 and remained elevated through d40 (Table 3; *p* ≤ 0.05), whereas *IGF2* increased between d10 and d20 before decreasing on d30 and returning to intermediate levels at d40 (Table 3; *p* ≤ 0.05). Hepatic expression of *IGFR1* exhibited a similar pattern to *IGF2* and went up between d10 and d20, was reduced on d30, and increased again on d40 (Table 3; *p* ≤ 0.05).

**FIGURE 4:**
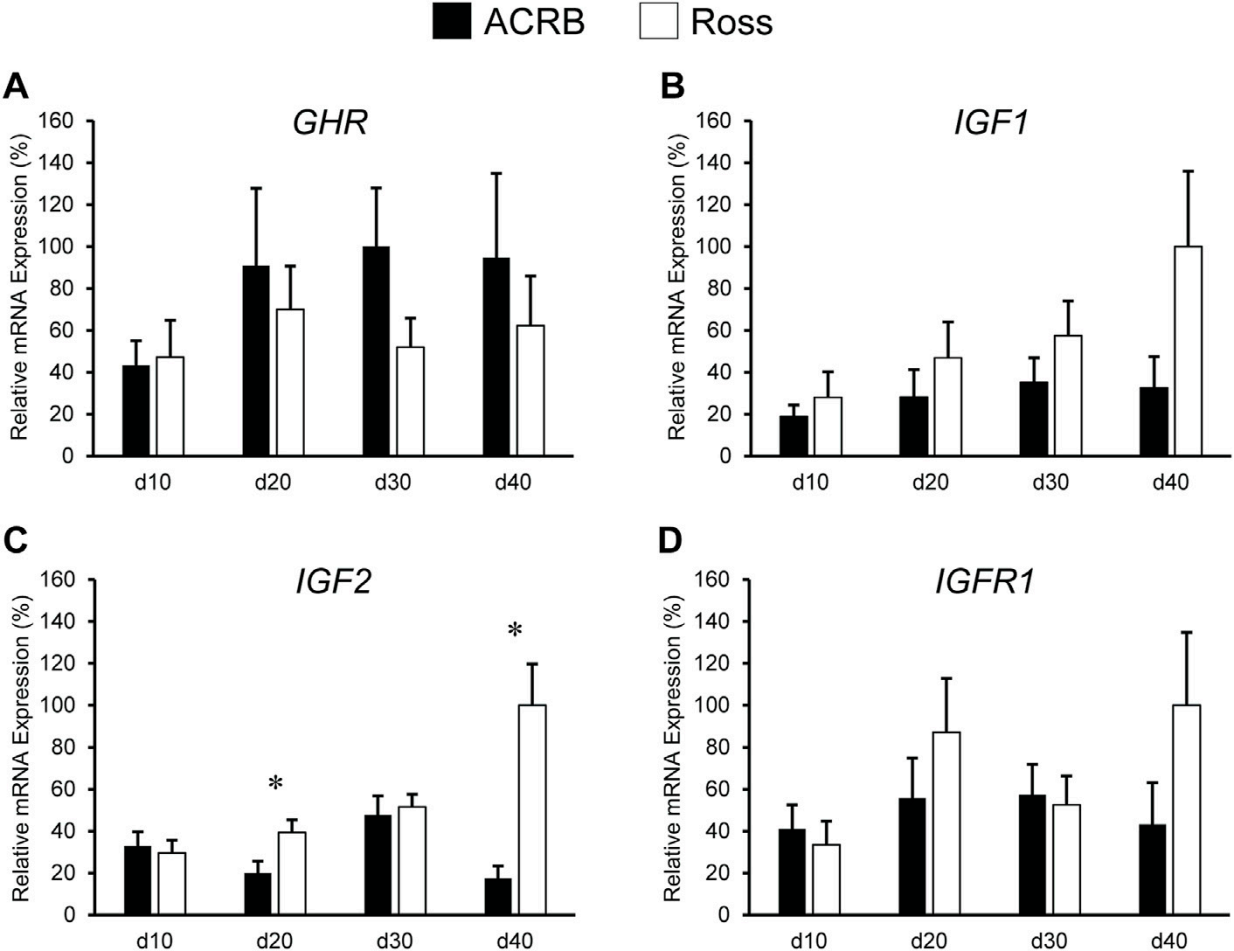
Relative mRNA expression of **(A)** *GHR*, **(B)** *IGF1*, **(C)** *IGF2*, and **(D)** *IGFR1* in breast muscle on post-hatch days d 10, 20, 30, and 40 in legacy ACRB and modern Ross 308 male broilers. Relative expression levels were measured using RT-qPCR and normalized to *18S* RNA (*n* = 8 replicate birds per line at each age). The data (mean + SEM) are expressed relative to the line and age with the highest expression level (equivalent to 100%). No significant line-by-age interactions were detected for **(A)** *GHR* (*p* = 0.5112), **(B)** *IGF1* (*p* = 0.1424), or **(D)** *IGF1R* (*p* = 0.1258), and main effect means of line and age for these genes are presented in Tables 4, 5, respectively. A significant line-by-age interaction was detected for **(C)** *IGF2* (*p* = 0.0111), and the presence of an asterisk (*) indicates a significant difference in expression between the lines at the indicated age.

Levels of these genes in post-hatch breast muscle are shown in Figure 4. No significant interactive effects were detected for *GHR* and *IGF1* (Figures 4A,B; *p* > 0.05), but each exhibited main line effects. Expression was higher overall in ACRB breast muscle for *GHR*, whereas *IGF1* mRNA levels were greater in Ross breast muscle (Table 4, *p* ≤ 0.05). *GHR* also displayed a main effect of age, increasing from d10 to d20 and remaining stable through d40 in this tissue (Table 4; *p* ≤ 0.05). Additionally, *IGF1* approached significance for a main effect of age, where breast muscle expression increased between d10 and d40 (Table 5; *p* = 0.0531). *IGF2* did demonstrate a significant line-by-age interactive effect, in which expression was two-fold greater in Ross breast muscle on d20 and increased to five-fold greater on d40 (Figure 4C; *p* ≤ 0.05). A significant interactive effect was not observed for *IGFR1* mRNA in breast muscle (Figure 4D; *p* > 0.05), but it approached significance for a main effect of age. Expression increased from d10 to d20, decreased at d30, and returned to d20 levels on d40 (Table 5; *p* = 0.0683).

**TABLE 4:** Means^1^ (±SEM) of the line main effect for gene expression and circulating hormones in post-hatch male broilers.

	ACRB	Ross 308	*p*-value
IGFs and Receptors			
Liver (%)^2^			
*IGF1*	100 ± 11.1	92.0 ± 10.5	0.6546
*IGF2*	100 ± 10.2	92.3 ± 10.1	0.4426
*IGF1R*	94.4 ± 7.3	100 ± 9.4	0.826
Muscle (%)^2^			
*GHR*	100 ± 18.2^a^	71.4 ± 11.4^b^	0.0447
*IGF1*	48.4 ± 9.4^b^	100 ± 20.2^a^	0.0009
*IGF1R*	71.1 ± 11.2	100 ± 17.8	0.242
IGFBPs			
Liver (%)^2^			
*IGFBP2*	100 ± 32.9^a^	67.9 ± 18.3^b^	0.0073
*IGFBP3*	83.4 ± 10.8^b^	100 ± 13.3^a^	0.0444
*IGFBP4*	92.8 ± 11.8	100 ± 15.7	0.9186
*IGFBP5*	69.0 ± 5.2^b^	100 ± 12.9^a^	0.0234
*IGFBP7*	66.3 ± 8.5^b^	100 ± 16.5^a^	0.0027
Muscle (%)^2^			
*IGFBP1*	100 ± 27.5	97.2 ± 38.7	0.3532
*IGFBP3*	100 ± 10.1^a^	70.08 ± 7.4^b^	0.0041
*IGFBP4*	54.1 ± 10.19^b^	100 ± 18.05^a^	0.0333
*IGFBP5*	60.6 ± 5.5^b^	100 ± 14.3^a^	0.0125
*IGFBP7*	75.2 ± 8.7^b^	100 ± 10.6^a^	0.0308
Hormones			
IGF1 (pg/ml)^3^	776.7 ± 21.5	796.7 ± 24.4	0.5014
IGF2 (pg/ml)^3^	190.9 ± 15.9	167.7 ± 19.8	0.7571

1 Means are only presented for data where a significant line-by-age interaction was not present and were calculated between post-hatch day 10 through 40 for each line.

2 Data within each gene are expressed relative to the line with the highest mRNA, level (100%).

3 Circulating hormone data are expressed as absolute concentration.

a,b Values that do not share a common letter are significantly different (*p* ≤ 0.05).

**TABLE 5:** Means^1^ (±SEM) of the age main effect for gene expression and circulating hormones in post-hatch male broilers.

	d10	d20	d30	d40	*p*-value
IGFs and Receptors					
Liver (%)^2^					
*IGF1*	26.6 ± 3.8^c^	72.0 ± 11.3^b^	90.6 ± 9.8^ab^	100 ± 9.9^a^	<0.0001
*IGF2*	52.9 ± 9.0^c^	100 ± 10.7^a^	53.5 ± 7.3^bc^	74.5 ± 10.5^ab^	0.007
*IGF1R*	65.1 ± 6.6^bc^	100 ± 6.9^a^	49.3 ± 4.6^c^	86.7 ± 11.7^ab^	0.0002
Muscle (%)^2^					
*GHR*	56.6 ± 12.4^b^	100 ± 24.8^a^	95.2 ± 20.3^a^	97.1 ± 27.7^ab^	0.0260
*IGF1*	33.7 ± 9.3	55.6 ± 15.9	67.6 ± 14.9	100 ± 31.6	0.0531
*IGF1R*	51.0 ± 10.7	98.6 ± 22.2	74.8 ± 13.1	100 ± 29.2	0.0683
IGFBPs					
Liver (%)^2^					
*IGFBP2*	3.4 ± 1.2^c^	100 ± 20.6^a^	10.4 ± 1.5^b^	14.1 ± 4.1^b^	<0.0001
*IGFBP3*	56.7 ± 11.6^b^	100 ± 15.6^a^	57.9 ± 8.5^b^	84.2 ± 16.6^a^	<0.0001
*IGFBP4*	18.1 ± 1.7^b^	92.1 ± 9.9^a^	86.9 ± 16.0^a^	100 ± 17.3^a^	<0.0001
*IGFBP5*	45.3 ± 6.6^c^	100 ± 7.4^a^	66.2 ± 4.24^b^	72.6 ± 19.9^b^	0.0006
*IGFBP7*	50.3 ± 13.3^c^	100 ± 20.0^a^	56.9 ± 6.3^b^	70.5 ± 18.2^b^	0.0393
Muscle (%)^2^					
*IGFBP1*	19.4 ± 6.5^c^	100 ± 41.9^a^	49.4 ± 22.3^ab^	42.4 ± 13.4^b^	0.0011
*IGFBP3*	68.4 ± 8.9	71.8 ± 9.3	100 ± 11.6	97.4 ± 18.5	0.1052
*IGFBP4*	19.4 ± 4.4^c^	30.4 ± 5.1^b^	75.7 ± 16.1^a^	100 ± 21.4^a^	<0.0001
*IGFBP5*	36.5 ± 3.6^b^	48.6 ± 3.4^b^	79.1 ± 9.5^a^	100 ± 21.5^a^	0.0003
*IGFBP7*	55.2 ± 6.5^b^	80.5 ± 12.6^a^	100 ± 13.8^a^	92.2 ± 15.9^a^	0.0029
Hormones					
IGF1 (pg/ml)^3^	698.3 ± 26.1^b^	798.3 ± 42.7^a^	811.3 ± 18.7^a^	839.8 ± 26.3^a^	0.0096
IGF2 (pg/ml)^3^	145.5 ± 13.8^b^	247.9 ± 27.5^a^	164.8 ± 21.5^b^	139.2 ± 23.6^b^	0.0042

1 Means are only presented for data where a significant line-by-age interaction was not present and were calculated across both lines at each post-hatch day (d).

2 Data within each gene are expressed relative to the age with the highest mRNA, level (100%).

3 Circulating hormone data are expressed as absolute concentration.

a,b,c Values that do not share a common letter are significantly different (*p* ≤ 0.05).

### Circulating Insulin-Like Growth Factors in Post-Hatch Plasma

**FIGURE 5:**
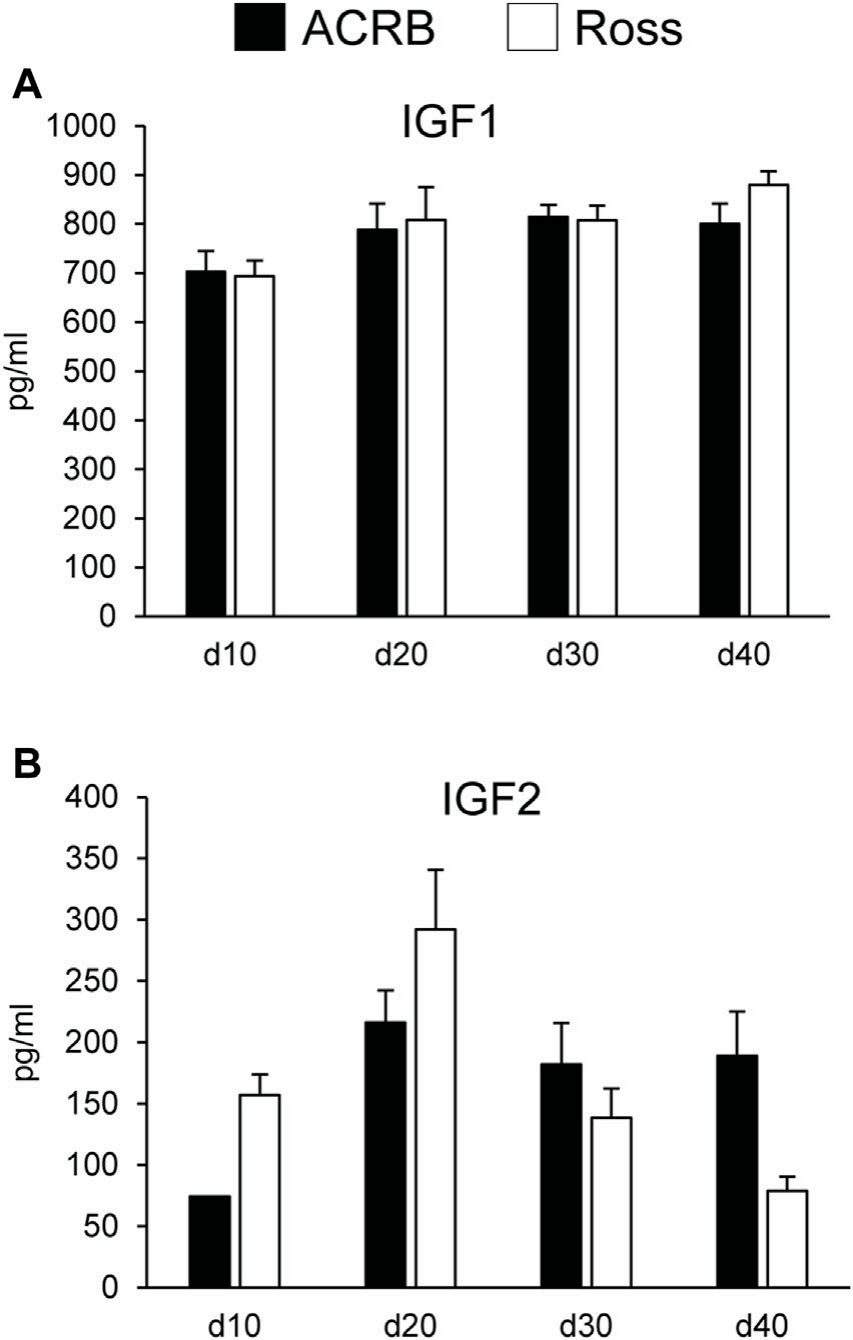
Circulating **(A)** IGF1 and **(B)** IGF2 in legacy ACRB and modern Ross 308 male broilers on post-hatch days d 10, 20, 30, and 40 as determined by ELISA (*n* = 8 replicate birds per line at each age). No significant line-by-age interactions were observed for **(A)** IGF1 (*p* = 0.7065) or **(B)** IGF2 (*p* = 0.0647), and main effect means of line and age are presented in Tables 4, 5, respectively.

Figure 5 shows circulating concentrations of IGF1 and IGF2 in post-hatch broilers, which were determined because of their ability to regulate overall body growth and induce cellular growth and proliferation in breast muscle. There was no significant line-by-age effect for IGF1 (Figure 5A; *p* > 0.05), although there was a main effect of age. Levels of IGF1 increased between d10 and d20 and remained elevated through d40 (Table 5; *p* ≤ 0.05). Circulating IGF2 approached significance for a line-by-age effect, in which IGF2 was greater in Ross at d10 and d20 but higher in ACRB on d40 (Figure 5B; *p* = 0.0647). IGF2 also exhibited a main effect of age, with circulating levels peaking on d20 in both lines (Table 5; *p* ≤ 0.05).

### Insulin-Like Growth Factor-Binding Protein Expression During Embryonic Development

**FIGURE 6:**
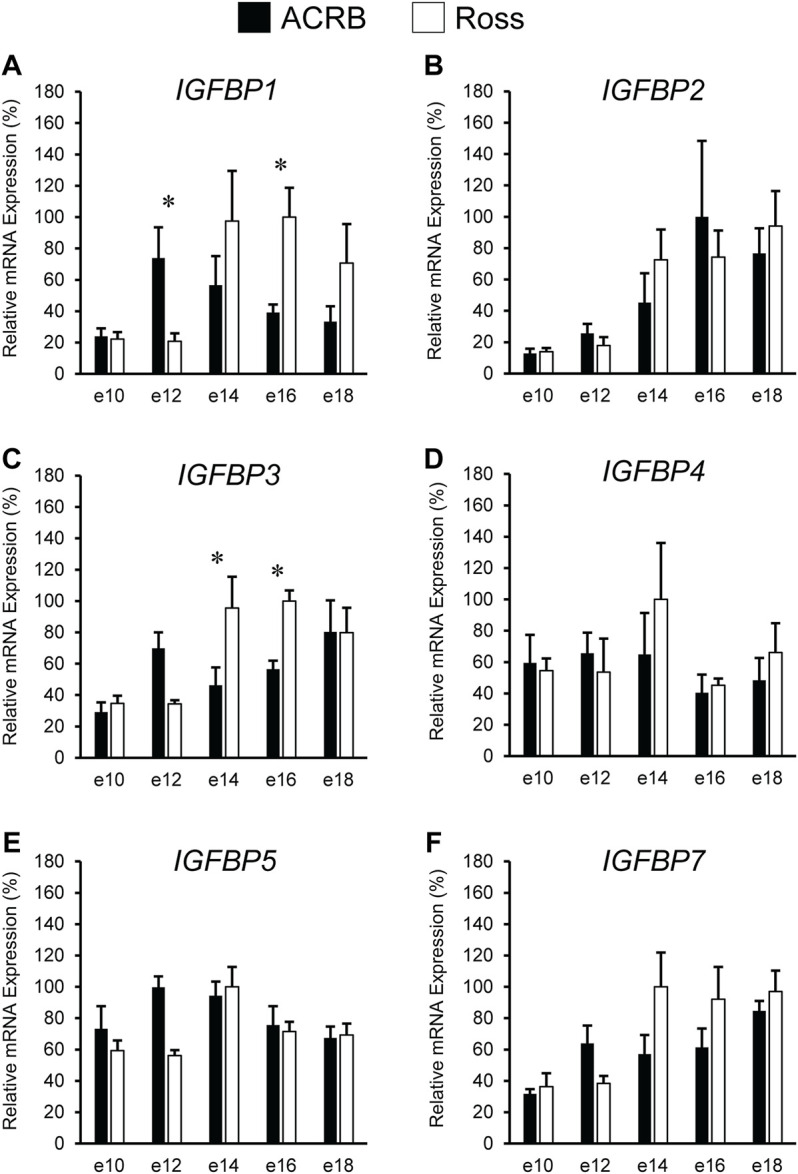
Relative mRNA expression of **(A)** *IGFBP1*, **(B)** *IGFBP2*, **(C)** *IGFBP3*, **(D)** *IGFBP4*, **(E)** *IGFBP5*, and **(F)** *IGFBP7* in liver on embryonic e days 10, 12, 14, 16, and 18 in legacy ACRB and modern Ross 308 male broilers. Relative expression levels were measured using RT-qPCR and normalized to *GAPDH* mRNA (*n* = 4 replicate birds per line at each age). The data (mean + SEM) are expressed relative to the line and age with the highest expression level (equivalent to 100%). Significant line-by-age interactions were detected for **(A)** *IGFBP1* (*p* = 0.0038) and **(C)** *IGFBP3* (*p* = 0.0080), and the presence of an asterisk (*) indicates a significant difference in expression between the lines at the indicated age (*p* ≤ 0.05). No significant line-by-age interactions were detected for **(B)** *IGFBP2* (*p* = 0.3060), **(D)** *IGFBP4* (*p* = 0.2942), **(E)** *IGFBP5* (*p* = 0.1055), or **(F)** *IGFBP7* (*p* = 0.0697), and main effect means of line and age for these genes are presented in Tables 2, 3, respectively.

The liver is a major producer of IGFBPs (Baxter, 1991), and this protein family is essential for controlling IGF signaling, thus regulates IGF effects on myogenic growth (Ewton et al., 1998; Kamanga-Sollo et al., 2005). Relative IGFBP expression levels measured in embryonic ACRB and Ross liver are presented in Figure 6. *IGFBP1* exhibited a significant line-by-age interaction, where ACRB expression at e12 was 4-fold greater than Ross but the opposite was observed at e16 when Ross expression was 2.5-fold greater than ACRB (Figure 6A; *p* ≤ 0.05). *IGFBP2* did not exhibit an interactive effect (Figure 6B; *p* > 0.05), but expression in liver was low from e10 to e12 and increased steadily thereafter through e18, indicating a main age effect (Table 3; *p* ≤ 0.05). *IGFBP3* exhibited a significant interactive effect and expression was approximately 2-fold greater in Ross liver than in ACRB liver on both e14 and e16 (Figure 6C; *p* ≤ 0.05). No interactive effects or main effects of line or age were observed for *IGFBP4* in this tissue (Figure 6D; Tables 2, 3; *p* ≤ 0.05). *IGFBP5* also did not have a significant interactive effect (Figure 6E; *p* > 0.05), but it approached significance for a main effect of line where hepatic ACRB expression was greater than that in Ross (Table 2; *p* = 0.094). Age was also significant for liver *IGFBP5* expression, increasing between e10 and e14 and decreasing on e16 and e18 (Table 5; *p* ≤ 0.05). *IGFBP7* displayed a nearly significant line-by-age interaction in embryonic liver (Figure 6F; *p* = 0.0697) and was greater in Ross than ACRB on e14. Additionally, its expression increased from e10 to e14, denoting a main effect of age (Table 3; *p* ≤ 0.05).

**FIGURE 7:**
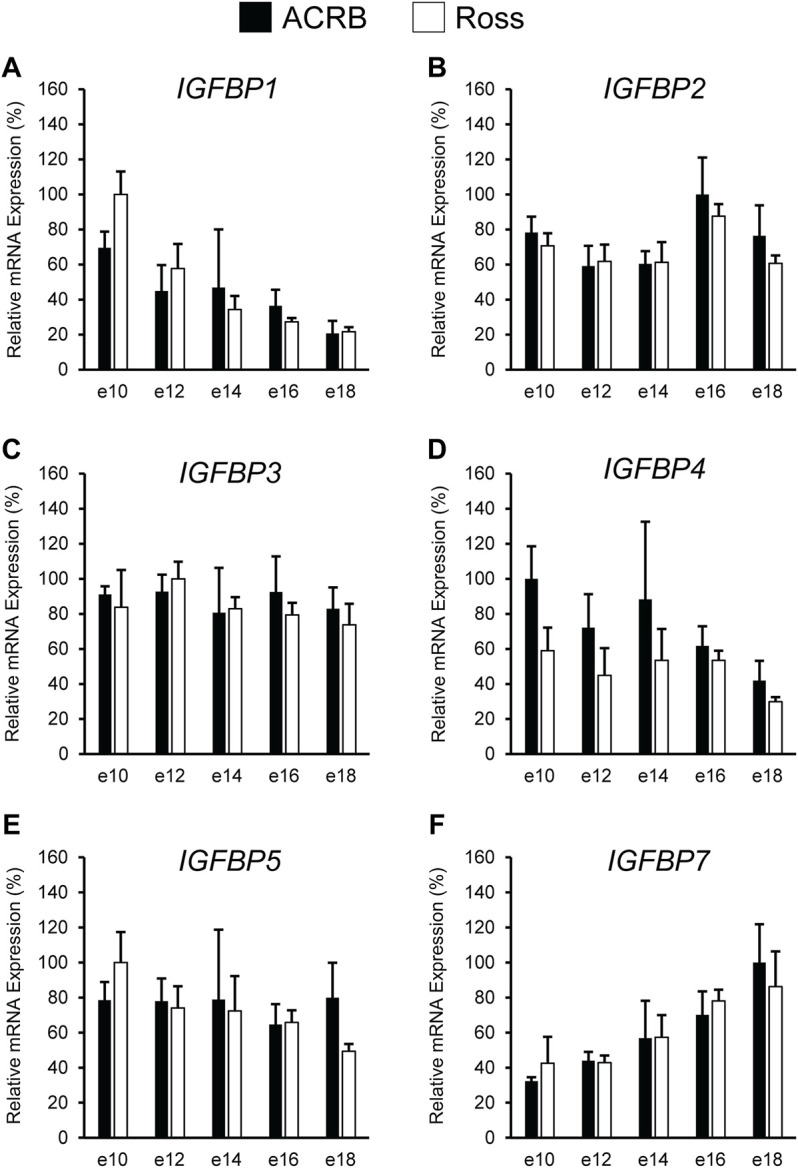
Relative mRNA expression of **(A)** *IGFBP1*, **(B)** *IGFBP2*, **(C)** *IGFBP3*, **(D)** *IGFBP4*, **(E)** *IGFBP5*, and **(F)** *IGFBP7* in breast muscle on embryonic days e 10, 12, 14, 16, and 18 in legacy ACRB and modern Ross 308 male broilers. Relative expression levels were measured using RT-qPCR and normalized to *18S* RNA (*n* = 4 replicate birds per line at each age). The data (mean + SEM) are expressed relative to the line and age with the highest expression level (equivalent to 100%). No significant line-by-age interactions were detected for **(A)** *IGFBP1* (*p* = 0.8032), **(B)** *IGFBP2* (*p* = 0.9609), **(C)** *IGFBP3* (*p* = 0.8806), **(D)** *IGFBP4* (*p* = 0.8715), **(E)** *IGFBP5* (*p* = 0.6831), or **(F)** *IGFBP7* (*p* = 0.9480), and main effect means of line and age for all genes are presented in Tables 2, 3, respectively.

The *IGFBPs* did not display any significant interactive effects in embryonic breast muscle (Figure 7; *p* > 0.05). *IGFBP1* and *IGFBP7* exhibited a main effect of age, with expression decreasing or increasing between e10 to e18, respectively (Table 3; *p* ≤ 0.05). No significant main effects of line or age were observed for *IGFBP2*, *IGFBP3*, or *IGFBP5* (Tables 2, 3; *p* > 0.05). A line main effect was detected for breast muscle *IGFBP4*, in which levels in Ross were significantly lower (Table 2; *p* ≤ 0.05).

### Insulin-Like Growth Factor-Binding Protein Expression During Post-Hatch Development

**FIGURE 8:**
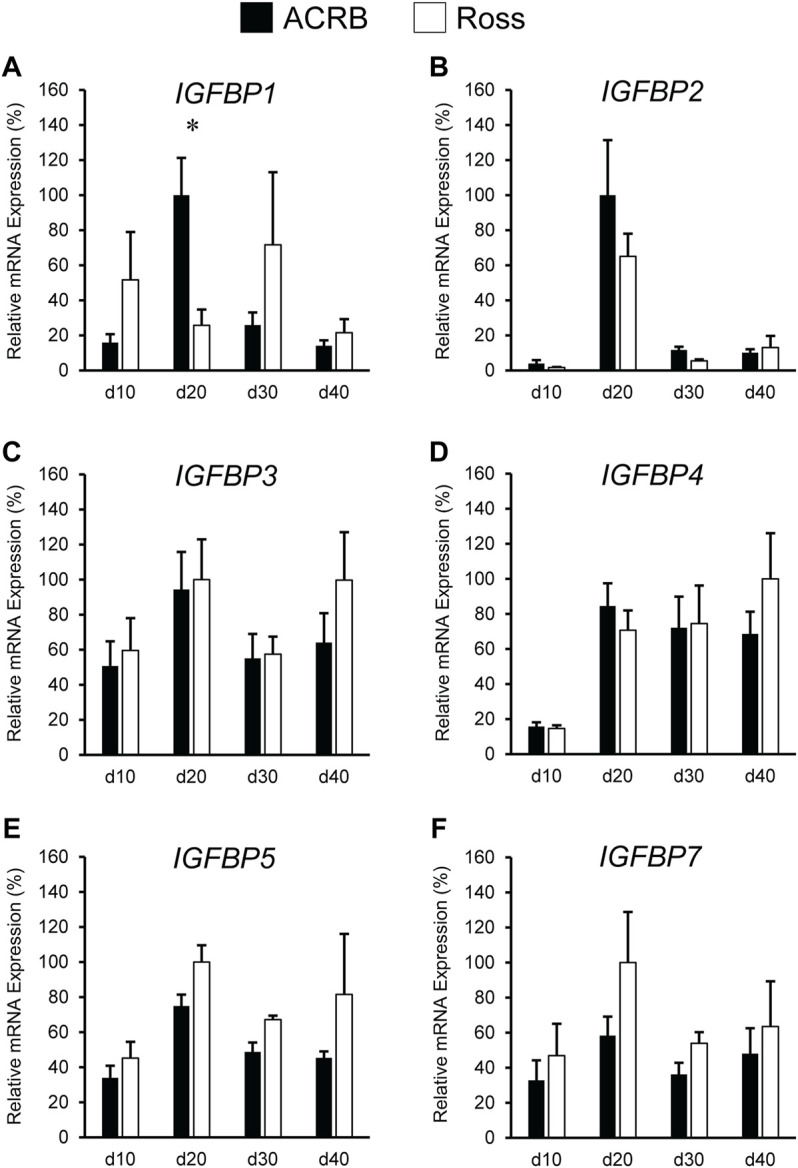
Relative mRNA expression of **(A)** *IGFBP1*, **(B)** *IGFBP2*, **(C)** *IGFBP3*, **(D)** *IGFBP4*, **(E)** *IGFBP5*, and **(F)** *IGFBP7* in liver on post-hatch days d 10, 20, 30, and 40 in legacy ACRB and modern Ross 308 male broilers. Relative expression levels were measured using RT-qPCR and normalized to *GAPDH* mRNA (*n* = 8 replicate birds per line at each age). The data (mean + SEM) are expressed relative to the line and age with the highest expression level (equivalent to 100%). A significant line-by-age interaction was detected for **(A)** *IGFBP1* (*p* = 0.0014), and the presence of an asterisk (*) indicates a significant difference in expression between the lines at the indicated age (*p* ≤ 0.05). No significant line-by-age interactions were detected for **(B)** *IGFBP2* (*p* = 0.5051), **(C)** *IGFBP3* (*p* = 0.5261), **(D)** *IGFBP4* (*p* = 0.5834), **(E)** *IGFBP5* (*p* = 0.8311), or **(F)** *IGFBP7* (*p* = 0.8716), and main effect means of line and age for these genes are presented in Tables 2, 3, respectively.

IGFBP expression in post-hatch liver is shown in Figure 8. Only *IGFBP1* exhibited a significant line-by-age interaction (Figure 8A; *p* ≤ 0.05), whereas the remaining IGFBPs did not (Figures 8B–F; *p* > 0.05). Levels of ACRB *IGFBP1* mRNA were 4-fold higher than Ross at d20 (Figure 7A; *p* ≤ 0.05) and numerically lower than Ross on d10 and d30. Main effects of line and age were observed for *IGFBP2* and *IGFBP3*, whereas *IGFBP4* only had a main effect of age. Liver expression of *IGFBP2* was greater in ACRB, while expression of *IGFBP3* was greater in Ross (Table 4; *p* ≤ 0.05). *IGFBP2* was 10- to 30-fold higher on d20 than other age, and *IGFBP3* expression on d20 and d40 was almost twice that of d10 and d30 (Table 5; *p* ≤ 0.05). After a 5-fold increase in expression between d10 and d20, *IGFBP4* remained high through d40 (Table 5; *p* ≤ 0.05). *IGFBP5* and *IGFBP7* also exhibited main effects of line and age. Expression of both genes were significantly greater in Ross liver (Table 4; *p* ≤ 0.05), and their expression increased approximately 2-fold between d10 and d20 and then decreased to intermediate levels of d30 and d40 (Table 5; *p* ≤ 0.05).

**FIGURE 9:**
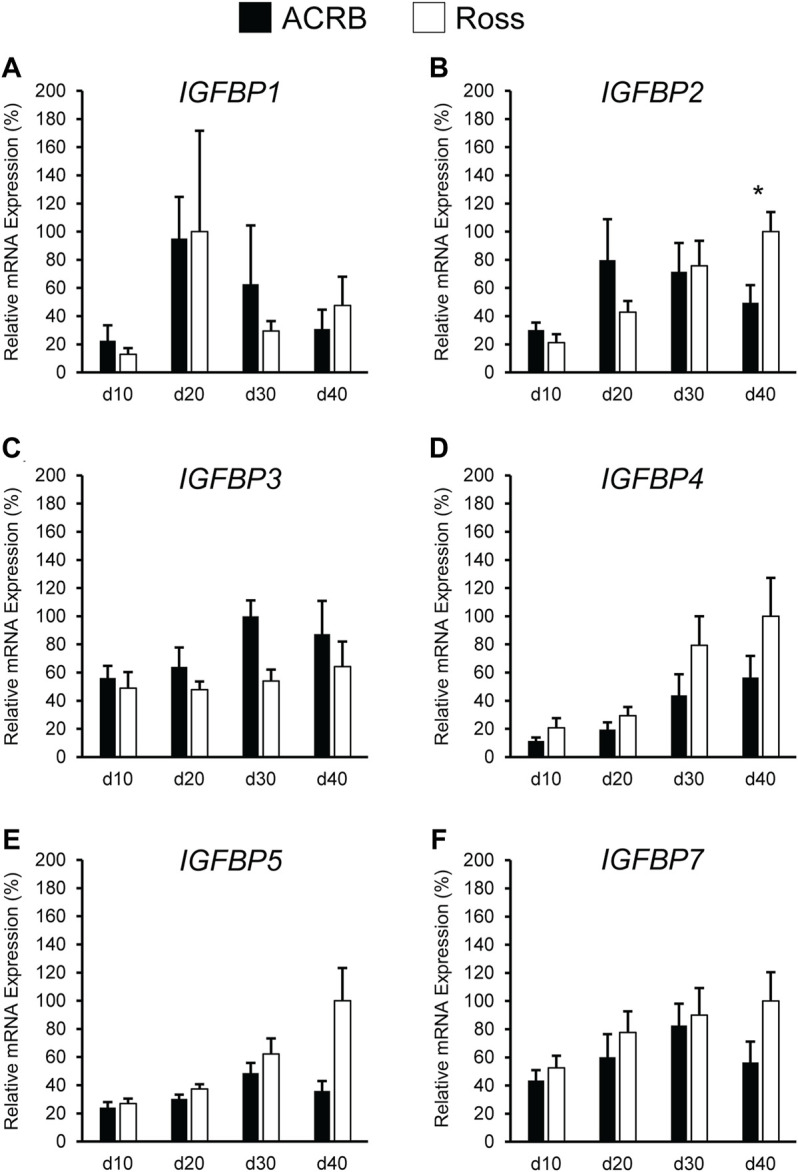
Relative mRNA expression of **(A)** *IGFBP1*, **(B)** *IGFBP2*, **(C)** *IGFBP3*, **(D)** *IGFBP4*, **(E)** *IGFBP5*, and **(F)** *IGFBP7* in breast muscle on post-hatch days d 10, 20, 30, and 40 in legacy ACRB and modern Ross 308 male broilers. Relative expression levels were measured using RT-qPCR and normalized to *18S* RNA (*n* = 8 replicate birds per line at each age). The data (mean + SEM) are expressed relative to the line and age with the highest expression level (equivalent to 100%). A significant line-by-age interaction was identified for **(B)** *IGFBP2* (*p* = 0.0022), and the presence of an asterisk (*) indicates a significant difference in expression between the lines at those ages (*p* ≤ 0.05). No significant line-by-age interactions were detected for **(A)** *IGFBP1* (*p* = 0.3093), **(C)** *IGFBP3* (*p* = 0.7127), **(D)** *IGFBP4* (*p* = 0.6558), **(E)** *IGFBP5* (*p* = 0.1711), or **(F)** *IGFBP7* (*p* = 0.4647), and main effect means of line and age for these genes are presented in Tables 4, 5, respectively.

Figure 9 illustrates IGFBP mRNA levels in post-hatch breast muscle. *IGFBP1* did not have a significant interactive effect (Figure 9A; *p* > 0.05) or line main effect (Table 4; *p* > 0.05) but did exhibit a main effect of age. Expression increased approximately 5-fold between d10 and d20 and was reduced about 2-fold at later ages (Table 5; *p* ≤ 0.05). *IGFBP2* displayed a significant line-by-age interaction in post-hatch breast muscle and was higher in Ross than ACRB at d40 (Figure 9B; *p* ≤ 0.05). No significant interactive effects were determined for *IGFBP3*, *IGFBP4*, *IGFBP5*, or *IGFBP7* (Figures 9C–F; *p* > 0.05)*,* but each demonstrated a main effect of line (Table 4; *p* ≤ 0.05). Apart from *IGFBP3*, which was higher in ACRB breast muscle, expression was greater in Ross (Table 4; *p* ≤ 0.05). Additionally, *IGFBP4*, *IGFBP5*, and *IGFBP7* expression differed significantly across ages. *IGFBP4* expression increased between d10 and d30 and remained high on d40 (Table 5; *p* ≤ 0.05). Levels of *IGFBP5* mRNA were lower at d10 and d20 than d30 and d40 (Table 5; *p* ≤ 0.05). Expression of *IGFBP7* increased significantly after d10 and remained high thereafter (Table 5; *p* ≤ 0.05).

## Discussion

The highly conserved nature of the somatotropic axis in vertebrates implies that it plays an important functional role in the growth and development of birds, though how it contributes to the improvements in growth rate and meat production efficiency made through artificial selection of commercial broilers is still not known. Thus, this study examined if components of the somatotropic axis, including hormones, hormone receptors, and hormone binding proteins, differed between a genetic control line (ACRB) and a modern commercial broiler line (Ross 308) during embryonic and post-hatch development. The results suggest that selection has impacted local IGF signaling in breast muscle more than endocrine action of circulating IGFs, and that IGFBPs play an important role in modulating somatotropic axis activity in a tissue-specific manner to affect growth. Multiple lines of evidence from this study suggest that classical somatotopic axis activity might not play a major role in driving chicken embryonic growth, in large part because embryonic IGF levels are likely not influenced by circulating GH. Pituitary GH in chickens increases during the last half of embryonic development (Porter et al., 1995; Ellestad et al., 2006; Lu et al., 2008; Parkinson et al., 2010; Ellestad et al., 2011), around the time that the birds used in this study began diverging in body weight. It was previously shown that Ross embryos were significantly heavier by e14, and body weight differences between the lines continued to increase through d40 (Vaccaro et al., 2021). In liver and breast muscle, neither *GHR* nor *IGF1* expression differed between the lines during embryonic development, suggesting that GH stimulation of IGF1 is not driving the observed differences in growth. While liver *IGF2* mRNA was higher in Ross 308 on e14, this was not maintained on e16 and 18 despite Ross embryos growing at a faster rate. *GHR* was observed to increase in liver and breast muscle during this period in both lines. However, this increase was accompanied by either no change or inconsistent changes in liver *IGF1*, *IGF2*, and *IGFR1* or a decrease in *IGF1* and *IGFR1* in breast muscle, suggesting that *IGF1*, *IGF2*, and *IGFR1* production are not dependent on GH during late embryonic development. It has been suggested that the somatotropic axis is not fully established until after hatch (Ellestad et al., 2011; Ellestad et al., 2019), and this study provides further evidence that IGF production is likely not GH-dependent in the embryonic somatotropic axis.

Heightened expression of *GHR* mRNA in liver and muscle throughout late embryonic development may be used for GH binding protein (GHBP) synthesis, which is made by cleaving off GHR’s extracellular domain (Vleurick et al., 1999; Lau et al., 2007). Human GHBPs form a complex with GH (Baumann et al., 1986), and this may similarly occur in chickens. As pituitary GH production increases late in chicken embryonic development, GHBP might sequester it until target tissues like liver and muscle are responsive to GH after the somatotropic axis is fully established.

It has been reported that pituitary and plasma GH levels are lower in fast-growing birds after hatch (Goddard et al., 1988; Mao et al., 1998; Ellestad et al., 2019). Hepatic *GHR* expression was greater in Ross than ACRB on d30 and d40, and this may reflect a need for increased GH sensitivity to compensate for reduced circulating GH relative to the slower-growing ACRB birds. This could be accomplished by providing additional plasma membrane binding sites for GH and/or by increasing its half-life in plasma *via* GHBP action. Ultimately, however, higher *GHR* in Ross liver does not appear to contribute to increased hepatic *IGF1* or *IGF2* expression or circulating IGF levels in relation to those parameters in to ACRB.

Levels of *IGF1* and *IGF2* mRNA were greater in post-hatch Ross breast muscle as compared to ACRB, suggesting these hormones support the rapid muscle growth observed in commercial modern broilers. Together with the observation that hepatic and circulating IGFs did not differ between the lines, these results indicate that differential paracrine IGF signaling may impact growth on a tissue-specific basis and contribute to the faster growth and increased muscle accretion in modern birds. Our findings align with the previously proposed theory that IGF signaling in chicken muscle acts in a paracrine fashion, contributing to hypertrophy in a manner similar to mice, rats, and rabbits (Czerwinski et al., 1994; Yang et al., 1997; Duclos et al., 1999).

The IGFBP family mediates IGF effects by enhancing or dampening IGF signaling. This occurs by either increasing IGF-receptor affinity, physically sequestering it to prevent receptor binding, or extending IGF’s half-life in circulation. Additionally, many IGFBPs can act independently to induce cellular activity (Kajimoto and Rotwein, 1989; Dewil et al., 1999; Herrington and Carter-Su, 2001; Woelfle et al., 2005; Brooks et al., 2008). Our results suggest that effects of some IGFBPs on broiler growth may differ between embryonic and post-hatch development. Expression of *IGFBP1* was greater in ACRB liver at e12 but increased in Ross liver at e16. This correlates with the difference in embryonic body weight between the lines previously observed beginning on e14 (Vaccaro et al., 2021). Here, elevated *IGFBP1* may serve to transport IGF in circulation, as liver *IGF2* in the embryo was greater in Ross at e10 and e14 and could facilitate growth during the last week of embryogenesis. In the liver of post-hatch ACRBs, however, *IGFBP1* was greater at d20, when broilers are growing most rapidly. Work performed in mice indicates *IGFBP1*, when produced in the liver, limits growth (Arany et al., 1994; Gay et al., 1997; Schneider et al., 2000), and it could act similarly in post-hatch chickens. Combined, these results indicate that *IGFBP1* function may change across developmental stages in broilers, in turn altering bird physiology by promoting IGF signaling during embryogenesis and inhibiting it during certain stages of juvenile post-hatch development.

IGFBPs function in an endocrine fashion when secreted into plasma from the liver but a paracrine one when produced locally in peripheral tissues (Allard and Duan, 2018). While levels of *IGFBP4* in liver did not differ between the lines at any stage, differential expression of *IGFBP4* in breast muscle suggests it may act locally to regulate growth of this tissue and, like *IGFBP1*, may have opposing effects during embryonic and post-hatch developmental stages. In embryonic development, elevated *IGFBP4* mRNA in ACRB breast muscle suggests in acts in an inhibitory manner. This would be consistent with previous reports that *IGFBP4* inhibited growth of mouse skeletal muscle (Jones and Clemmons, 1995; Awede et al., 1999). The effect in breast muscle is likely to be IGF-dependent, because *IGFBP4* inhibits cellular proliferation of myoblasts only in the presence of *IGF1* (Ewton et al., 1998). Since expression of *IGF1* and *IGF2* mRNA in breast muscle did not differ between the lines, it is possible that elevated *IGFBP4* in ACRB reduces IGF signaling in this tissue through its sequestration. On the other hand, during post-hatch development, *IGFBP4* appears to act in a paracrine manner to stimulate breast muscle growth. Levels of *IGFBP4* mRNA in Ross breast muscle post-hatch were almost twice that of ACRB, as were *IGF1* and *IGF2* mRNA. This indicates that, in post-hatch breast muscle, *IGFBP4* could work to perpetuate IGF signaling through increasing the hormones’ half-life and/or facilitating their access to *IGFR1*.

*IGFBP7* may also regulate skeletal muscle generation in chickens based on results presented here. *IGFBP7* has been shown to limit cell cycle activation in mice, protecting against satellite cell exhaustion to ensure long-term muscle growth (Chen et al., 2020). Increased *IGFBP7* mRNA was observed in Ross broiler breast muscle post-hatch, suggesting it could work in a similar manner to promote muscle growth after hatch by maintaining a healthy satellite cell population. This could contribute to greater breast muscle yield in commercial modern broilers (Schmidt et al., 2009; Collins et al., 2014; Marks et al., 2016) by supporting the satellite cell population and facilitating their differentiation during muscle accretion.

Within the same developmental stage, the effects of a singular IGFBP can also change depending on whether it acts in an endocrine or paracrine manner. Hepatic post-hatch *IGFBP2* was greater in ACRB, aligning with inhibitory *IGFBP2* action observed in zebrafish where it reduced cell proliferation during fasting (Duan et al., 1999). However, *IGFBP2* was greater in post-hatch Ross breast muscle later in development. Since *IGFBP2* has been shown to induce chicken primary myoblast proliferation (Wang et al., 2019), this might mean that endocrine *IGFBP2* released from post-hatch liver inhibits overall body growth but paracrine *IGFBP2* activity in breast muscle facilitates its growth. Data presented here suggest that the inverse may be true for *IGFBP3*, which has a promotive effect on IGF signaling in mammals when acting in an endocrine manner by extending their half-life in the blood (Yamada and Lee, 2009) but may inhibit breast muscle growth by acting in paracrine manner. *IGFBP3* mRNA was greater in Ross embryonic liver at e14 and e16, ages at which they start increasing in size relative to ACRBs. Thus, when synthesized in the liver, *IGFBP3* could extend IGF signaling by maintaining IGFs in the blood of Ross embryos and contribute to their larger size that begins around late embryogenesis. Importantly, elevated hepatic *IGFBP3* in Ross birds continued post-hatch, playing into its established role as a metabolic regulator (Yamada et al., 2010) and suggesting it may also impact body composition and feed efficiency in chickens. Post-hatch *IGFBP3* was reduced in Ross muscle compared to ACRB, suggesting that it may negatively regulate muscle accretion through direct sequestration of IGFs or in another manner. Together, these results are indicative that IGFBPs act in a tissue-specific manner to control IGF signaling through both endocrine and paracrine mechanisms and can have both inhibitory and stimulatory effects depending on their mode of action, as has been observed in mammals.

Like *IGFBP3*, hepatic *IGFBP5* and *IGFBP7* mRNA levels were higher in post-hatch Ross broilers, indicative of an endocrine effect by these proteins that promotes bird growth and muscle accretion. In mice, it was shown that single knockouts for *IGFBP3*, *IGFBP4*, or *IGFBP5* showed little growth impairment, while triple knockout mice were significantly smaller with reduced fat pad accumulation and less skeletal muscle (Ning et al., 2006). This indicates that some IGFBPs exhibit functional redundancy in regulating growth and metabolism in mammals, and a similar phenomenon might exist in birds.

To summarize, we found that expression levels of select somatotropic genes differed between male legacy and commercial modern broilers. Although there were no differences in circulating IGFs, elevated *IGF1* and *IGF2* in post-hatch Ross muscle suggests that paracrine IGF signaling contributes to the increased breast muscle size of commercial modern broilers. Control of IGF signaling by IGFBPs likely also differs between commercial modern and legacy broilers and plays a role in regulating chicken growth. It was observed that select IGFBPs appear to play distinct, and sometimes opposing. growth-promoting or growth-inhibiting roles in a developmental and tissue-specific manner and that functional redundancy among the IGFBPs may exist. In conclusion, these results suggest that rapid growth and increased muscle accretion in commercial modern broilers may be achieved not through increased levels of circulating IGFs but by changing local IGF expression to affect paracrine IGF activity, specifically in muscle. This activity could be further regulated through combinatorial action of IGFBPs, which appear to make up a robust control system acting to support growth within different developmental and physiological contexts.

## Data Availability

The raw data supporting the conclusion of this article will be made available by the authors, without undue reservation.

## References

[B1] Allander S. V. Ehrenborg E. Luthman H. Powell D. R. (1995). Conservation of IGFBP Structure during Evolution: Cloning of Chicken Insulin-like Growth Factor Binding Protein-5. Prog. Growth Factor Res. 6 (2), 159–165. 10.1016/0955-2235(96)00011-7 8817657

[B2] Allander S. V. Coleman M. Luthman H. Powell D. R. (1997). Chicken Insulin-like Growth Factor Binding Protein (IGFBP)-5: Conservation of IGFBP-5 Structure and Expression during Evolution. Comp. Biochem. Physiology Part B Biochem. Mol. Biol. 116 (4), 477–483. 10.1016/S0305-0491(96)00289-1 9149401

[B3] Allard J. B. Duan C. (2018). IGF-binding Proteins: Why Do They Exist and Why Are There So Many? Front. Endocrinol. 9, 117. 10.3389/fendo.2018.00117 PMC5900387 29686648

[B4] Arany E. Afford S. Strain A. J. Winwood P. J. Arthur M. J. Hill D. J. (1994). Differential Cellular Synthesis of Insulin-like Growth Factor Binding Protein-1 (IGFBP-1) and IGFBP-3 within Human Liver. J. Clin. Endocrinol. Metabolism 79 (6), 1871–1876. 10.1210/jcem.79.6.7527416 7527416

[B5] Armstrong D. G. McKay C. O. Morrell D. J. Goddard C. (1989). Insulin-like Growth Factor-I Binding Proteins in Serum from the Domestic Fowl. J. Endocrinol. 120 (3), 373–378. 10.1677/joe.0.1200373 2466933

[B6] Awede B. Thissen J.-P. Gailly P. Lebacq J. (1999). Regulation of IGF-I, IGFBP-4 and IGFBP-5 Gene Expression by Loading in Mouse Skeletal Muscle. FEBS Lett. 461 (3), 263–267. 10.1016/S0014-5793(99)01469-6 10567708

[B7] Bartov I. (1982). Corticosterone and Fat Deposition in Broiler Chicks: Effect of Injection Time, Breed, Sex and Age. Br. Poult. Sci. 23 (2), 161–170. 10.1080/00071688208447942 7074385

[B8] Baumann G. Stolar M. W. Amburn K. Barsano C. P. DeVries B. C. (1986). A Specific Growth Hormone-Binding Protein in Human Plasma: Initial Characterization*. J. Clin. Endocrinol. Metabolism 62 (1), 134–141. 10.1210/jcem-62-1-134 3940261

[B9] Baxter R. C. (1991). Insulin-like Growth Factor (IGF) Binding Proteins: the Role of Serum IGFBPs in Regulating IGF Availability. Acta Paediatr. 80 (s372), 107–114. 10.1111/j.1651-2227.1991.tb17983.x 1718141

[B10] Beccavin C. Chevalier B. Cogburn L. Simon J. Duclos M. (2001). Insulin-like Growth Factors and Body Growth in Chickens Divergently Selected for High or Low Growth Rate. J. Endocrinol. 168 (2), 297–306. 10.1677/joe.0.1680297 11182767

[B11] Berrong S. L. Washburn K. W. (1998). Effects of Genetic Variation on Total Plasma Protein, Body Weight Gains, and Body Temperature Responses to Heat Stress. Poult. Sci. 77 (3), 379–385. 10.1093/ps/77.3.379 9521447

[B12] Brooks A. J. Wooh J. W. Tunny K. A. Waters M. J. (2008). Growth Hormone Receptor; Mechanism of Action. Int. J. Biochem. Cell Biol. 40 (10), 1984–1989. 10.1016/j.biocel.2007.07.008 17888716

[B13] Burnside J. Cogburn L. A. (1992). Developmental Expression of Hepatic Growth Hormone Receptor and Insulin-like Growth Factor-I mRNA in the Chicken. Mol. Cell Endocrinol. 89 (1), 91–96. 10.1016/0303-7207(92)90214-Q 1301387

[B14] Burnside J. Liou S. S. Zhong C. Cogburn L. A. (1992). Abnormal Growth Hormone Receptor Gene Expression in the Sex-Linked Dwarf Chicken. General Comp. Endocrinol. 88 (1), 20–28. 10.1016/0016-6480(92)90190-U 1426960

[B15] Buyse J. Decuypere E. (1999). The Role of the Somatotrophic axis in the Metabolism of the Chicken. Domest. Anim. Endocrinol. 17 (2), 245–255. 10.1016/S0739-7240(99)00041-7 10527127

[B16] Chen C. Chen Y. H. Tixier-Boichard M. Cheng P. Y. Chang C. S. Tang P.-C. (2009). Effects of the Chicken Sex-Linked Dwarf Gene on Growth and Muscle Development. Asian-australas. J. Anim. Sci. 22. 10.5713/ajas.2009.80689

[B17] Chen Z. Li L. Wu W. Liu Z. Huang Y. Yang L. (2020). Exercise Protects Proliferative Muscle Satellite Cells against Exhaustion via the Igfbp7-Akt-mTOR axis. Theranostics 10 (14), 6448–6466. 10.7150/thno.43577 32483463 PMC7255041

[B18] Clark R. Robinson I. C. (1996). Up and Down the Growth Hormone Cascade. Cytokine & Growth Factor Rev. 7 (1), 65–80. 10.1016/1359-6101(96)00006-8 8864355

[B19] Collins K. E. Kiepper B. H. Ritz C. W. McLendon B. L. Wilson J. L. (2014). Growth, Livability, Feed Consumption, and Carcass Composition of the Athens Canadian Random Bred 1955 Meat-type Chicken versus the 2012 High-Yielding Cobb 500 Broiler. Poult. Sci. 93 (12), 2953–2962. 10.3382/ps.2014-04224 25352681

[B20] Collins K. E. Marks H. L. Aggrey S. E. Lacy M. P. Wilson J. L. (2016). History of the Athens Canadian Random Bred and the Athens Random Bred Control Populations. Poult. Sci. 95 (5), 997–1004. 10.3382/ps/pew085 26976904

[B21] Czerwinski S. M. Cate J. M. Francis G. Tomas F. Brocht D. M. McMurtry J. P. (1998). The Effect of Insulin-like Growth Factor-I (IGF-I) on Protein Turnover in the Meat-type Chicken (*Gallus domesticus*). Comp. Biochem. Physiology Part C Pharmacol. Toxicol. Endocrinol. 119 (1), 75–80. 10.1016/s0742-8413(97)00193-x 9568376

[B22] Czerwinski S. M. Martin J. M. Bechtel P. J. (1994). Modulation of IGF mRNA Abundance during Stretch-Induced Skeletal Muscle Hypertrophy and Regression. J. Appl. Physiology 76 (5), 2026–2030. 10.1152/jappl.1994.76.5.2026 8063665

[B23] D'Costa A. P. Prevette D. M. Houenou L. J. Wang S. Zackenfels K. Rohrer H. (1998). Mechanisms of Insulin-like Growth Factor Regulation of Programmed Cell Death of Developing Avian Motoneurons. J. Neurobiol. 36 (3), 379–394. 10.1002/(sici)1097-4695(19980905)36:3<379::aid-neu6>3.0.co;2-t 9733073

[B24] Dewil E. Darras V. M. Spencer G. S. G. Lauterio T. J. Decuypere E. (1999). The Regulation of GH-dependent Hormones and Enzymes after Feed Restriction in Dwarf and Control Chickens. Life Sci. 64 (16), 1359–1371. 10.1016/S0024-3205(99)00082-X 10321716

[B25] Duan C. Ding J. Li Q. Tsai W. Pozios K. (1999). Insulin-like Growth Factor Binding Protein 2 Is a Growth Inhibitory Protein Conserved in Zebrafish. Proc. Natl. Acad. Sci. U.S.A. 96 (26), 15274–15279. 10.1073/pnas.96.26.15274 10611375 PMC24810

[B26] Duclos M. J. Beccavin C. Simon J. (1999). Genetic Models for the Study of Insulin-like Growth Factors (IGF) and Muscle Development in Birds Compared to Mammals. Domest. Anim. Endocrinol. 17 (2), 231–243. 10.1016/S0739-7240(99)00040-5 10527126

[B27] Duclos M. J. Goddard C. (1990). Insulin-like Growth Factor Receptors in Chicken Liver Membranes: Binding Properties, Specificity, Developmental Pattern and Evidence for a Single Receptor Type. J. Endocrinol. 125 (2), 199–206. 10.1677/joe.0.1250199 2165118

[B28] Ellestad L. E. Carre W. Muchow M. Jenkins S. A. Wang X. Cogburn L. A. (2006). Gene Expression Profiling during Cellular Differentiation in the Embryonic Pituitary Gland Using cDNA Microarrays. Physiol. Genomics 25 (3), 414–425. 10.1152/physiolgenomics.00248.2005 16493019

[B29] Ellestad L. E. Cogburn L. A. Simon J. Le Bihan-Duval E. Aggrey S. E. Byerly M. S. (2019). Transcriptional Profiling and Pathway Analysis Reveal Differences in Pituitary Gland Function, Morphology, and Vascularization in Chickens Genetically Selected for High or Low Body Weight. BMC Genomics 20 (1), 316. 10.1186/s12864-019-5670-9 31023219 PMC6482517

[B30] Ellestad L. E. Malkiewicz S. A. Guthrie H. D. Welch G. R. Porter T. E. (2009). Expression and Regulation of Glucocorticoid-Induced Leucine Zipper in the Developing Anterior Pituitary Gland. J. Mol. Endocrinol. 42 (2), 171–183. 10.1677/jme-08-0066 19060181

[B31] Ellestad L. E. Porter T. E. (2013). Ras-dva Is a Novel Pit-1- and Glucocorticoid-Regulated Gene in the Embryonic Anterior Pituitary Gland. Endocrinology 154 (1), 308–319. 10.1210/en.2012-1566 23161868 PMC3591683

[B32] Ellestad L. E. Puckett S. A. Porter T. E. (2015). Mechanisms Involved in Glucocorticoid Induction of Pituitary GH Expression during Embryonic Development. Endocrinology 156 (3), 1066–1079. 10.1210/en.2014-1686 25560830 PMC4330307

[B33] Ellestad L. E. Saliba J. Porter T. E. (2011). Ontogenic Characterization of Gene Expression in the Developing Neuroendocrine System of the Chick. General Comp. Endocrinol. 171 (1), 82–93. 10.1016/j.ygcen.2010.12.006 21168412

[B34] Ewton D. Z. Coolican S. A. Mohan S. Chernausek S. D. Florini J. R. (1998). Modulation of Insulin-like Growth Factor Actions in L6A1 Myoblasts by Insulin-like Growth Factor Binding Protein (IGFBP)-4 and IGFBP-5: a Dual Role for IGFBP-5. J. Cell. Physiol. 177 (1), 47–57. 10.1002/(sici)1097-4652(199810)177:1<47::aid-jcp5>3.0.co;2-e 9731744

[B35] Fisher M. C. Meyer C. Garber G. Dealy C. N. (2005). Role of IGFBP2, IGF-I and IGF-II in Regulating Long Bone Growth. Bone 37 (6), 741–750. 10.1016/j.bone.2005.07.024 16183342

[B36] Fridolfsson A.-K. Ellegren H. (1999). A Simple and Universal Method for Molecular Sexing of Non-ratite Birds. J. Avian Biol. 30 (1), 116–121. 10.2307/3677252

[B37] Frommer K. W. Reichenmiller K. Schutt B. S. Hoeflich A. Ranke M. B. Dodt G. (2006). IGF-independent Effects of IGFBP-2 on the Human Breast Cancer Cell Line Hs578T. J. Mol. Endocrinol. 37 (1), 13–23. 10.1677/jme.1.01955 16901920

[B38] Frost R. A. Lang C. H. (1999). Differential Effects of Insulin-like Growth Factor I (IGF-I) and IGF-Binding Protein-1 on Protein Metabolism in Human Skeletal Muscle Cells1. Endocrinology 140 (9), 3962–3970. 10.1210/endo.140.9.6998 10465265

[B39] Gahete M. D. Luque R. M. Castaño J. P. (2016). Models of GH Deficiency in Animal Studies. Best Pract. Res. Clin. Endocrinol. Metabolism 30 (6), 693–704. 10.1016/j.beem.2016.11.001 27974185

[B40] Gay E. Seurin D. Babajko S. Doublier S. Cazillis M. Binoux M. (1997). Liver-Specific Expression of Human Insulin-like Growth Factor Binding Protein-1 in Transgenic Mice: Repercussions on Reproduction, Ante- and Perinatal Mortality and Postnatal Growth1. Endocrinology 138 (7), 2937–2947. 10.1210/endo.138.7.5282 9202238

[B41] Giachetto P. F. Riedel E. C. Gabriel J. E. Ferro M. I. T. Di Mauro S. M. Z. Macari M. (2004). Hepatic mRNA Expression and Plasma Levels of Insulin-like Growth Factor-I (IGF-I) in Broiler Chickens Selected for Different Growth Rates. Genet. Mol. Biol. 27, 39–44. 10.1590/S1415-47572004000100007

[B42] Girbau M. Bassas L. Alemany J. de Pablo F. (1989). *In Situ* autoradiography and Ligand-dependent Tyrosine Kinase Activity Reveal Insulin Receptors and Insulin-like Growth Factor I Receptors in Prepancreatic Chicken Embryos. Proc. Natl. Acad. Sci. U.S.A. 86 (15), 5868–5872. 10.1073/pnas.86.15.5868 2548191 PMC297732

[B43] Goddard C. Wilkie R. S. Dunn I. C. (1988). The Relationship between Insulin-like Growth Factor-1, Growth Hormone, Thyroid Hormones and Insulin in Chickens Selected for Growth. Domest. Anim. Endocrinol. 5 (2), 165–176. 10.1016/0739-7240(88)90017-3 3066582

[B44] Havenstein G. B. Ferket P. R. Scheideler S. E. Larson B. T. (1994). Growth, Livability, and Feed Conversion of 1957 vs 1991 Broilers when Fed “Typical” 1957 and 1991 Broiler Diets. Poult. Sci. 73 (12), 1785–1794. 10.3382/ps.0731785 7877934

[B45] Havenstein G. Ferket P. Qureshi M. (2003). Growth, Livability, and Feed Conversion of 1957 versus 2001 Broilers when Fed Representative 1957 and 2001 Broiler Diets. Poult. Sci. 82 (10), 1500–1508. 10.1093/ps/82.10.1500 14601725

[B46] Herrington J. Carter-Su C. (2001). Signaling Pathways Activated by the Growth Hormone Receptor. Trends Endocrinol. Metab. 12 (6), 252–257. 10.1016/S1043-2760(01)00423-4 11445442

[B47] Hess C. W. (1962). Randombred Populations of the Southern Regional Poultry Breeding Project. World's Poult. Sci. J. 18 (2), 147–152. 10.1079/WPS19620019

[B48] Hutt F. B. (1959). Sex-Linked Dwarfism in the Fowl. J. Hered. 50 (5), 209–221. 10.1093/oxfordjournals.jhered.a106909

[B49] Huybrechts L. M. Decuypere E. Buyse J. Kühn E. R. Tixier-Boichard M. (1992). Effect of Recombinant Human Insulin-like Growth Factor-I on Weight Gain, Fat Content, and Hormonal Parameters in Broiler Chickens. Poult. Sci. 71 (1), 181–187. 10.3382/ps.0710181 1539018

[B50] Jones J. I. Clemmons D. R. (1995). Insulin-Like Growth Factors and Their Binding Proteins: Biological Actions*. Endocr. Rev. 16 (1), 3–34. 10.1210/edrv-16-1-3 7758431

[B51] Kajimoto Y. Rotwein P. (1989). Structure and Expression of a Chicken Insulin-like Growth Factor I Precursor. Mol. Endocrinol. 3 (12), 1907–1913. 10.1210/mend-3-12-1907 2628728

[B52] Kamanga-Sollo E. Pampusch M. S. White M. E. Hathaway M. R. Dayton W. R. (2005). Insulin-like Growth Factor Binding Protein (IGFBP)-3 and IGFBP-5 Mediate TGF-β- and Myostatin-Induced Suppression of Proliferation in Porcine Embryonic Myogenic Cell Cultures. Exp. Cell Res. 311 (1), 167–176. 10.1016/j.yexcr.2005.09.003 16214131

[B53] Kelley K. Schmidt K. Berg L. Sak K. Galima M. Gillespie C. (2002). Comparative Endocrinology of the Insulin-like Growth Factor-Binding Protein. J. Endocrinol. 175 (1), 3–18. 10.1677/joe.0.1750003 12379486

[B54] Kim J. W. (2010). The Endocrine Regulation of Chicken Growth. Asian Australas. J. Anim. Sci. 23 (12), 1668–1676. 10.5713/ajas.2010.10329

[B55] Lau J. S. Yip C. W. Law K. M. Leung F. C. (2007). Cloning and Characterization of Chicken Growth Hormone Binding Protein (cGHBP). Domest. Anim. Endocrinol. 33 (1), 107–121. 10.1016/j.domaniend.2006.04.012 16814975

[B56] Levine J. E. (2012). “An Introduction to Neuroendocrine Systems,” in Handbook of Neuroendocrinology. Editors Fink G. Pfaff D. W. Levine J. E. (San Diego: Academic Press), 3–19. 10.1016/b978-0-12-375097-6.10001-0

[B57] Livak K. J. Schmittgen T. D. (2001). Analysis of Relative Gene Expression Data Using Real-Time Quantitative PCR and the 2−ΔΔCT Method. Methods 25 (4), 402–408. 10.1006/meth.2001.1262 11846609

[B58] Lu F. Z. Wang X. X. Pan Q. X. Huang R. H. Liu H. L. (2008). Expression of Genes Involved in the Somatotropic, Thyrotropic, and Corticotropic Axes during Development of Langshan and Arbor Acres Chickens. Poult. Sci. 87 (10), 2087–2097. 10.3382/ps.2007-00493 18809871

[B59] Mao J. Burnside J. Postel-Vinay M. Pesek J. Cogburn L. Cogburn L. A. (1998). Ontogeny of Growth Hormone Receptor Gene Expression in Tissue of Growth-Selected Strains of Broiler Chickens. J. Endocrinol. 156 (1), 67–75. 10.1677/joe.0.1560067 9496235

[B60] McGuinness M. C. Cogburn L. A. (1991). Response of Young Broiler Chickens to Chronic Injection of Recombinant-Derived Human Insulin-like Growth Factor-I. Domest. Anim. Endocrinol. 8 (4), 611–620. 10.1016/0739-7240(91)90031-E 1786708

[B61] Mohan S. Nakao Y. Honda Y. Landale E. Leser U. Dony C. (1995). Studies on the Mechanisms by Which Insulin-like Growth Factor (IGF) Binding Protein-4 (IGFBP-4) and IGFBP-5 Modulate IGF Actions in Bone Cells. J. Biol. Chem. 270 (35), 20424–20431. 10.1074/jbc.270.35.20424 7544787

[B62] Ning Y. Schuller A. G. P. Bradshaw S. Rotwein P. Ludwig T. Frystyk J. (2006). Diminished Growth and Enhanced Glucose Metabolism in Triple Knockout Mice Containing Mutations of Insulin-like Growth Factor Binding Protein-3, -4, and -5. Mol. Endocrinol. 20 (9), 2173–2186. 10.1210/me.2005-0196 16675541

[B63] Parkinson N. Collins M. M. Dufresne L. Ryan A. K. (2010). Expression Patterns of Hormones, Signaling Molecules, and Transcription Factors during Adenohypophysis Development in the Chick Embryo. Dev. Dyn. 239 (4), 1197–1210. 10.1002/dvdy.22250 20175188

[B64] Payne J. A. Proszkowiec-Weglarz M. Ellestad L. E. (2019). Delayed Access to Feed Alters Expression of Genes Associated with Carbohydrate and Amino Acid Utilization in Newly Hatched Broiler Chicks. Am. J. Physiology-Regulatory, Integr. Comp. Physiology 317 (6), R864–R878. 10.1152/ajpregu.00117.2019 PMC6962625 31596116

[B65] Porter T. E. Couger G. S. Dean C. E. Hargis B. M. (1995). Ontogeny of Growth Hormone (GH)-secreting Cells during Chicken Embryonic Development: Initial Somatotrophs Are Responsive to GH-Releasing Hormone. Endocrinology 136 (5), 1850–1856. 10.1210/endo.136.5.7720629 7720629

[B66] Porter T. E. (1998). Differences in Embryonic Growth Hormone Secretion between Slow and Fast Growing Chicken Strains. Growth Hormone IGF Res. 8 (2), 133–139. 10.1016/S1096-6374(98)80103-2 10987680

[B67] Reiprich K. Mühlbauer E. Decuypere E. Grossmann R. (1995). Characterization of Growth Hormone Gene Expression in the Pituitary and Plasma Growth Hormone Concentrations during Posthatch Development in the Chicken. J. Endocrinol. 145 (2), 343–353. 10.1677/joe.0.1450343 7616168

[B68] Rutledge R. G. Stewart D. (2008). Critical Evaluation of Methods Used to Determine Amplification Efficiency Refutes the Exponential Character of Real-Time PCR. BMC Mol. Biol. 9 (1), 96. 10.1186/1471-2199-9-96 18973660 PMC2587475

[B69] Scanes C. G. Dunnington E. A. Buonomo F. C. Donoghue D. J. Siegel P. B. (1989). Plasma Concentrations of Insulin like Growth Factors (IGF-)I and IGF-II in Dwarf and Normal Chickens of High and Low Weight Selected Lines. Growth Dev. Aging 53 (4), 151–157. 2638344

[B70] Schmidt C. J. Persia M. E. Feierstein E. Kingham B. Saylor W. W. (2009). Comparison of a Modern Broiler Line and a Heritage Line Unselected since the 1950s. Poult. Sci. 88 (12), 2610–2619. 10.3382/ps.2009-00055 19903960

[B71] Schneider M. R. Lahm H. Wu M. Hoeflich A. Wolf E. (2000). Transgenic Mouse Models for Studying the Functions of Insulin‐like Growth Factor‐binding Proteins. FASEB J. 14 (5), 629–640. 10.1096/fasebj.14.5.629 10744620

[B72] Schoen T. J. Bondy C. A. Zhou J. Dhawan R. Mazuruk K. Arnold D. R. (1995). Differential Temporal and Spatial Expression of Insulin-like Growth Factor Binding Protein-2 in Developing Chick Ocular Tissues. Invest. Ophthalmol. Vis. Sci. 36 (13), 2652–2662. 7499087

[B73] Schutt B. Langkamp M. Rauschnabel U. Ranke M. Elmlinger M. (2004). Integrin-mediated Action of Insulin-like Growth Factor Binding Protein-2 in Tumor Cells. J. Mol. Endocrinol. 32 (3), 859–868. 10.1677/jme.0.0320859 15171717

[B74] Stewart C. E. Rotwein P. (1996). Growth, Differentiation, and Survival: Multiple Physiological Functions for Insulin-like Growth Factors. Physiol. Rev. 76 (4), 1005–1026. 10.1152/physrev.1996.76.4.1005 8874492

[B75] Vaccaro L. A. Porter T. E. Ellestad L. E. (2022). Effects of Genetic Selection on Activity of Corticotropic and Thyrotropic Axes in Modern Broiler Chickens. Domest. Anim. Endocrinol. 78, 106649. 10.1016/j.domaniend.2021.106649 34418578

[B76] Vleurick L. Kühn E. R. Decuypere E. Burnside J. Pezet A. Edery M. (1999). Generation of Chicken Growth Hormone-Binding Proteins by Proteolysis. General Comp. Endocrinol. 113 (2), 283–289. 10.1006/gcen.1998.7202 10082631

[B77] Wang Z. Zhang X. Li Z. Abdalla B. A. Chen Y. Nie Q. (2019). MiR-34b-5p Mediates the Proliferation and Differentiation of Myoblasts by Targeting IGFBP2. Cells 8 (4), 360. 10.3390/cells8040360 PMC6523632 30999686

[B78] Woelfle J. Chia D. J. Massart-Schlesinger M. B. Moyano P. Rotwein P. (2005). Molecular Physiology, Pathology, and Regulation of the Growth Hormone/insulin-like Growth Factor-I System. Pediatr. Nephrol. 20 (3), 295–302. 10.1007/s00467-004-1602-1 15549418

[B79] Yamada P. M. Lee K.-W. (2009). Perspectives in Mammalian IGFBP-3 Biology: Local vs. Systemic Action. Am. J. Physiology-Cell Physiology 296 (5), C954–C976. 10.1152/ajpcell.00598.2008 19279229

[B80] Yamada P. M. Mehta H. H. Hwang D. Roos K. P. Hevener A. L. Lee K. W. (2010). Evidence of a Role for Insulin-like Growth Factor Binding Protein (IGFBP)-3 in Metabolic Regulation. Endocrinology 151 (12), 5741–5750. 10.1210/en.2010-0672 20926583 PMC2999488

[B81] Yang S. Alnaqeeb M. Simpson H. Goldspink G. (1997). Changes in Muscle Fibre Type, Muscle Mass and IGF-I Gene Expression in Rabbit Skeletal Muscle Subjected to Stretch. J. Anat. 190 (4), 613–622. 10.1046/j.1469-7580.1997.19040613.x 9183683 PMC1467645

